# Building a translational cancer dependency map for The Cancer Genome Atlas

**DOI:** 10.1038/s43018-024-00789-y

**Published:** 2024-07-15

**Authors:** Xu Shi, Christos Gekas, Daniel Verduzco, Sakina Petiwala, Cynthia Jeffries, Charles Lu, Erin Murphy, Tifani Anton, Andy H. Vo, Zhiguang Xiao, Padmini Narayanan, Bee-Chun Sun, Aloma L. D’Souza, J. Matthew Barnes, Somdutta Roy, Cyril Ramathal, Michael J. Flister, Zoltan Dezso

**Affiliations:** 1 AbbVie Bay Area, South San Francisco, CA USA; 2 AbbVie, North Chicago, IL USA; 3 Pfizer Oncology Research and Development, Bothell, WA USA

**Keywords:** Cancer genomics, Machine learning, Target identification

## Abstract

Cancer dependency maps have accelerated the discovery of tumor vulnerabilities that can be exploited as drug targets when translatable to patients. The Cancer Genome Atlas (TCGA) is a compendium of ‘maps’ detailing the genetic, epigenetic and molecular changes that occur during the pathogenesis of cancer, yet it lacks a dependency map to translate gene essentiality in patient tumors. Here, we used machine learning to build translational dependency maps for patient tumors, which identified tumor vulnerabilities that predict drug responses and disease outcomes. A similar approach was used to map gene tolerability in healthy tissues to prioritize tumor vulnerabilities with the best therapeutic windows. A subset of patient-translatable synthetic lethalities were experimentally tested, including *PAPSS1*/*PAPSS12* and *CNOT7*/*CNOT78*, which were validated in vitro and in vivo. Notably, *PAPSS1* synthetic lethality was driven by collateral deletion of *PAPSS2* with *PTEN* and was correlated with patient survival. Finally, the translational dependency map is provided as a web-based application for exploring tumor vulnerabilities.

## Abstract (web summary)

Shi et al. present a hybrid dependency map based on machine-learning analysis of gene essentiality data from the DEPMAP database, translated to data from TCGA. This application can be used to visualize other gene essentiality data.

## Main

The rapid expansion of genomic technologies to characterize healthy and diseased patient populations has provided unprecedented resolution to the pathophysiological drivers of cancer and many other diseases. In 2018, TCGA completed a 10-year study of 33 tumor types across ~11,000 patients, which has broadly illuminated the genetic underpinnings of cancer^1^. Building on the success of TCGA, multiple other initiatives have been launched to explore aspects of cancer initiation, evolution, metastasis and response to therapy^2–6^, with the hope that the deepening molecular characterization of cancer will improve diagnosis, treatment and prevention; however, a critical step toward fully leveraging patient data to eradicate cancer is to assign functionality to the observations made in TCGA that translate putative tumor dependencies to life-saving therapies.

One approach to understanding tumor dependencies is through genome-wide genetic and chemical perturbation datasets (for example, DEPMAP^7,8^, Project SCORE^9^ and Connectivity Map^10^) that have been paired with thousands of deeply characterized cancer models (for example, Cancer Cell Line Encyclopedia^11^, Cancer Cell Line Factory^12^ and Human Cancer Models Initiative^13^). Multiple studies have demonstrated the ability of DEPMAP to translate gene essentially to therapeutic targets^14–18^ and a broader functional understanding of tumor dependencies^19,20^. Compared to TCGA, a differentiating strength of the ‘dependency maps’ is that hypotheses can be readily tested, replicated and refined in different contexts, whereas patient datasets are typically not amenable to functional experimentation; however, the dependency maps also pose limitations when compared to the translatability of TCGA, as homogeneous cell lines in culture dishes do not replicate the pathophysiological complexities of the intact tumor microenvironment^21^. Further, the current experimental models do not completely recapitulate the genetic drivers that are present in the patient population^22^, and experimental outcomes of genetic perturbation screens do not capture most aspects of disease outcome and patient survival.

To address the unique challenges posed by TCGA and DEPMAP, we built a hybrid dependency map (TCGA_DEPMAP_) via machine learning of gene essentiality in the cell-based DEPMAP that was then translated to TCGA patient tumors. As such, TCGA_DEPMAP_ leverages the experimental strengths of DEPMAP, while enabling patient-relevant translatability of TCGA. A systematic analysis of TCGA_DEPMAP_ revealed tumor vulnerabilities that predicted treatment response and patient outcomes, including lineage dependencies, oncogenes and synthetic lethalities. The flexible machine-learning framework was also used to assemble maps that captured other aspects of patient-relevant features, including translating dependencies to drug responses in the Patient-Derived Xenograft (PDX) Encyclopedia (PDXE_DEPMAP_) and tolerability within healthy tissues of the Genotype-Tissue Expression project (GTEX_DEPMAP_). Combined with a user-friendly and freely available web-based application, these data provide a resource for identifying patient-relevant tumor vulnerabilities that can be exploited as drug targets.

## Results

### Predictive modeling of gene essentiality

**Fig. 1:**
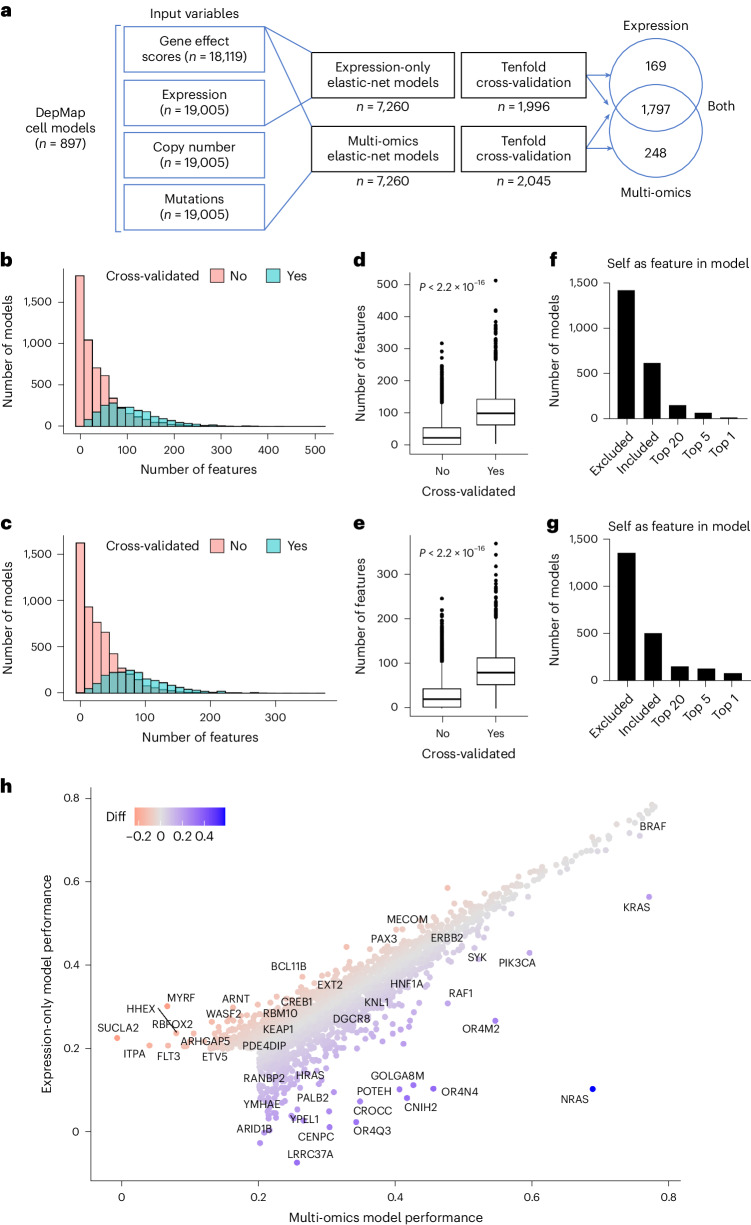
Predictive modeling of gene essentiality in the DEPMAP. **a**, Schematic of the elastic-net models for predictive modeling of gene essentiality in the DEPMAP using expression-only data or multi-omics data. Note the broad overlap in cross-validated models using expression-only or multi-omics data. **b**, Distribution of the number features per multi-omics model. **c**, Distribution of the number of features per expression-only model. **d**, Number of features per multi-omics model that passed (*n* = 2,045) or failed (*n* = 5,215) cross-validation based on a correlation coefficient of 0.2 threshold. **e**, Number of features per expression-only model that passed (*n* = 1,966) or failed (5,294) cross-validation based on a correlation coefficient of 0.2 threshold. For **d** and **e**, the center horizontal line represents the median (50th percentile) value. The box spans from the 25th to the 75th percentile. The whiskers indicate the fifth and 95th percentiles. **f**, Rank of the target gene (self) as a feature in the cross-validated multi-omics models. **g**, Rank of the target gene (self) as a feature in the cross-validated expression-only models. **h**, Comparison of model performance (correlation coefficients) of cross-validated models from multi-omics and expression-only data. Note for **b**–**h** that the performance and characteristics of multi-omics and expression-only models are very similar. *P* values indicated on graphs were determined by the Wilcoxon rank-sum test for two-group comparison (**d** and **e**). Source data

To begin building the translational dependency maps, predictive models of gene essentiality were trained on genome-wide CRISPR-Cas9 knockout screens from the DEPMAP^8^ using elastic-net regularization for feature selection and modeling^23^ (Fig. 1a). Genome-wide gene essentiality scores for DEPMAP cancer cell models (*n* = 897) were estimated by CERES^24^, which measures the essentiality of each gene relative to the distribution of effect sizes for common essential and nonessential genes within each cell line^25^. Because many genes do not impact cell viability, elastic-net models were attempted only for genes with at least five dependent and nondependent cell lines, which included 7,260 out of 18,119 genes (40%) with gene essentiality scores in the DEPMAP. In addition to gene essentiality scores, the input variables for elastic-net predictive modeling included genome-wide gene expression, mutation and copy number profiles for each cancer cell model. Based on previous evidence that predictive modeling of gene essentiality with RNA expression performed comparably to similar modeling that also included DNA features^26,27^, two sets of elastic-net models were compared using RNA alone (expression only) or combined with mutation and copy number profiles (multi-omics). Finally, the best fitting elastic-net models were selected by a tenfold cross-validation to identify models with the minimum error, while balancing the predictive performance with the number of features selected (Methods).

**Extended Data Fig. 1:**
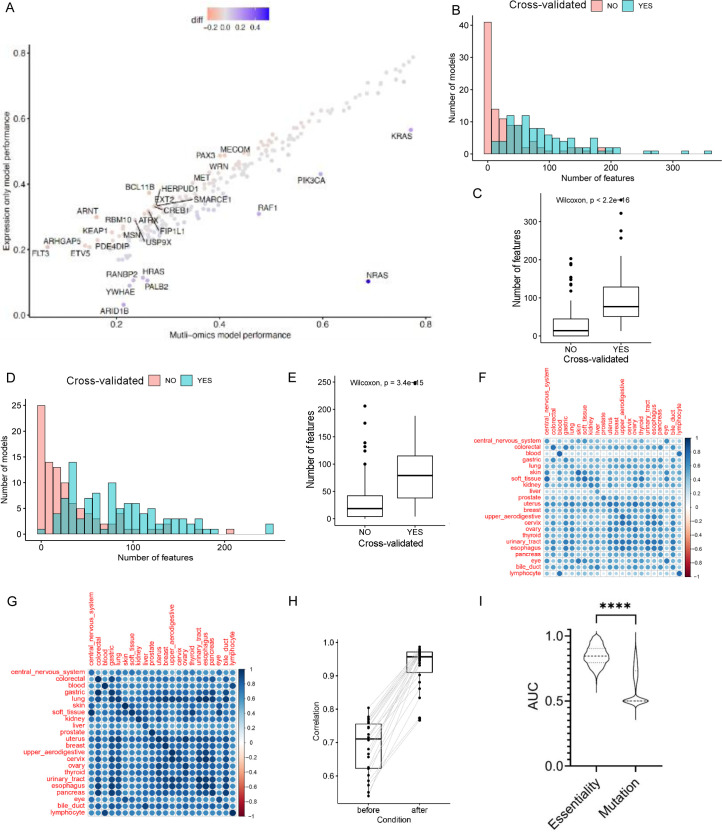
The characteristics of gene essentiality models before and after transcriptional alignment cell models and patient tumor biopsies. (**a**) The performances of expression-only and multi-omics models of gene essentiality were compared across 103 annotated oncogenes. Note the strong correlation of expression-only and multi-omics models with a few notable outliers, such as *NRAS, FLT3* and *ARNT*. (**b**) The distribution of the number of features for the multi-omics models for the 103 annotated oncogenes. (**c**) The number of features per multi-omics model for the 103 annotated oncogenes that passed (n = 95) or failed (n = 102) cross-validation. (**d**) The distribution of the number of features per expression-only models for the 103 annotated oncogenes. (**e**) The number of features per expression-only model for the 103 annotated oncogenes that passed (n = 101) or failed (n = 96) cross-validation. Note similarities in the characteristics and performances of multi-omics and expression-only models, and that only 7% of the multi-omics models significantly outperformed the expression-only models in the cross-validation while 84% were comparable when applying a cutoff of 0.05 correlation coefficient difference between models as a meaningful improvement in performance. As a reference using the same criteria 15% of multi-omics models outperformed expression-based models and 76% were comparable when we used the whole set of 2,211 models. (**f**, **g**) The heatmaps show the Pearson correlation between the gene expression of DepMap and TCGA before (**f**) and after (**g**) expression alignment by identification and removal of the most variant signatures (cPC1–4; that is, stromal signatures) before elastic-net ML. The rows are TCGA lineages and columns are DepMap lineages. (**h**) Shows that the correlation of expression for the same lineage (n = 22) in TCGA and DepMap is significantly improved by our expression alignment pipeline. (**i**) Comparison of expression-only elastic-net models for gene essentiality and gene mutational status (n = 890). To make performance metrics (AUC) comparable with binary mutational status, the essentiality scores were binarized using a –0.5 essentiality score as a cutoff. To calculate the accuracy of predicting dependencies and mutations, elastic-net machine learning was run to predict mutations and essentiality using the same settings and expression data for 891 genes with mutations at >2% prevalence in TCGA_DEPMAP_ patients. Of note, the elastic-net models were allowed to select the most informative predictive features for mutation and essentiality for each gene, as the best predictors for essentiality may not be the best features to predict mutation. For (C,E,H,I), the center horizontal line represents the median (50th percentile) value. The box spans from the 25th to the 75th percentile. The whiskers indicate the 5th and 95th percentiles. The two-sided Wilcoxon rank test was used for (C,E,H) and for (I) ****P < 0.0001 by Student unpaired t-test. Source data

The elastic-net models for predicting essentiality of the 7,260 genes (as described above) were compared by tenfold cross-validation (Pearson’s *r* > 0.2; false discovery rate (FDR) < 1 × 10^−3^) when considering expression-only or multi-omics data as input variables (Supplementary Tables 1 and 2). The distribution of features per model skewed higher in the multi-omics models (3–510 features, median of 98) (Fig. 1b) compared to the expression-only models (3–369 features, median of 80) (Fig. 1c) and the performance of both improved with the number of features per model (Fig. 1d,e). Of the 7,260 models, cross-validation confirmed 1,966 expression-only models and 2,045 multi-omics models, of which most cross-validated models overlapped (*n* = 1,797) (Supplementary Table 3). The incidence of self-inclusion of the target gene in the cross-validated models was also similar between multi-omics dataset (31% of models) (Fig. 1f) and expression-only dataset (26% of models) (Fig. 1g). The majority of cross-validated models (76%) performed comparably (within a correlation coefficient of 0.05) using either expression-only or multi-omics data. Likewise, 86 out of 103 annotated oncogenes (84%) with cross-validated models performed similarly using either expression-only or multi-omics datasets (for example, *HER2*, *BRAF* and *PIK3CA*), with a few notable examples that included the oncogenes: *NRAS*, *FLT3* and *ARNT* (Fig. 1h and Extended Data Fig. 1a–e). Collectively, these data demonstrate that predictive models of gene essentiality with expression-only (Supplementary Table 1) and multi-omics (Supplementary Table 2) data as input variables perform comparably in detecting selective vulnerabilities of cancer in most cases (Supplementary Table 3).

### Constructing TCGA_DEPMAP_

TCGA_DEPMAP_ was built using the expression-only elastic-net models of gene essentiality, based on the evidence here (Fig. 1) and elsewhere^26,27^ that the performance of most models was comparable to those including genomic features. Moreover, as genetic information is withheld from the expression-only elastic-net models, the transposed essentiality scores can be correlated with genetic drivers in TCGA_DEPMAP_ patients who might otherwise be missed in cancer cell models. Finally, expression-based predictive modeling of essentiality can also be extended to non-oncological studies (for example, GTEX), which do not have somatic mutations and copy number changes^28^.

**Fig. 2:**
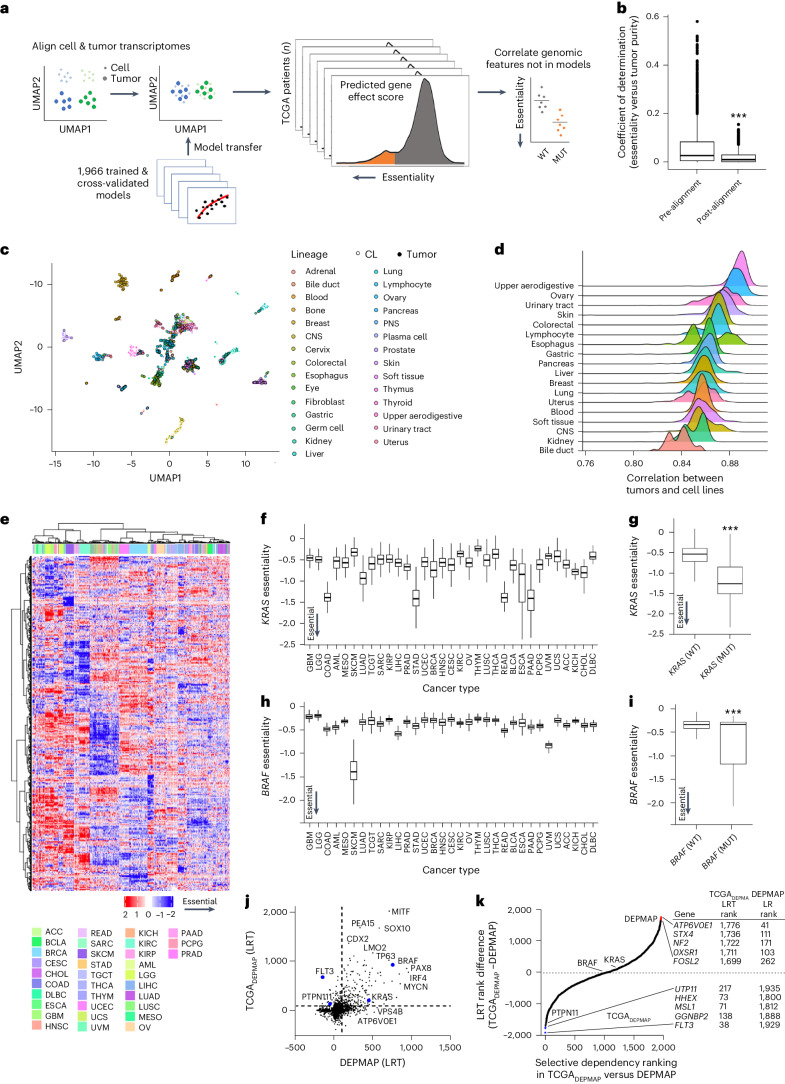
Building a translational dependency map: TCGA_DEPMAP_. **a**, Schematic of gene essentiality model transposition from DEPMAP to TCGA, following alignment of genome-wide expression data to account for differences in homogeneous cultured cell lines and heterogenous tumor biopsies with stroma. **b**, Coefficient of determination (*R*^2^) of the cross-validated gene essentiality models and tumor purity before (*n* = 1,966) and after transcriptional alignment (*n* = 1,966). The center horizontal line represents the median (50th percentile) value. The box spans from the 25th to the 75th percentile. The whiskers indicate the fifth and 95th percentiles. A two-sided Wilcoxon rank-sum test was performed to test for statistical significance. **c**, Uniform Manifold Approximation and Projection (UMAP) visualization of normalization of genome-wide transcriptomes improves alignment between cultured cells and patient tumor biopsies with contaminating stroma. **d**, Correlation coefficients of essentiality profiles of different lineages of cultured cell models and TCGA patient tumors. **e**, Unsupervised clustering of predicted gene essentiality scores across TCGA_DEPMAP_ revealed strong lineage dependencies. Blue indicates genes with stronger essentiality and red indicates genes with less essentiality. **f**, *KRAS* dependency was enriched in TCGA_DEPMAP_ lineages (*n* = 9,593) with high frequency of *KRAS* GOF mutations, including colon adenocarcinoma (COAD), LUAD, STAD, READ, esophageal carcinoma (ESCA) and PAAD. **g**, *KRAS* essentiality correlated with *KRAS* mutations in all TCGA_DEPMAP_ lineages (*n* = 532 for *KRAS*^mut^ and *n* = 7,049 for *KRAS*^wt^). **h**, *BRAF* dependency in TCGA_DEPMAP_ (*n* = 9,593) was enriched in SKCM, which has a high frequency of GOF mutations in *BRAF*. **i**, *BRAF* essentiality correlated with *BRAF* mutations in all TCGA_DEPMAP_ lineages (*n* = 559 for *BRAF*^mut^ and *n* = 7,022 for *BRAF*^wt^). For **f**–**i**, the center horizontal line represents the median (50th percentile) value. The box spans from the 25th to the 75th percentile. The whiskers indicate the fifth and 95th percentiles. For **g**–**i**, a two-sided Wilcoxon rank-sum test was performed to test for statistical significance. **j**, Scatter-plot of model selectivity in TCGA_DEPMAP_ and DEPMAP, as determined by normality likelihood (NormLRT). **k**, Ranking of model selectivity between in TCGA_DEPMAP_ and DEPMAP, as determined by the NormLRT scores. ***P < 0.001, as determined by the Wilcoxon rank-sum test for two-group comparison and Kruskal–Wallis followed by Wilcoxon rank-sum test with multiple test correction for the multi-group comparison. CNS, central nervous system; PNS, peripheral nervous system; ACC, adrenocortical carcinoma; BLCA, bladder urothelial carcinoma; CESC, cervical and endocervical cancers; CHOL, cholangiocarcinoma; GBM, glioblastoma multiforme; HNSC, head and neck squamous cell carcinoma; KIRC, kidney renal clear cell carcinoma; KIRP, kidney renal papillary cell carcinoma; LGG, lower-grade glioma; LIHC, liver hepatocellular carcinoma; MESO, mesothelioma; OV, ovarian serous cystadenocarcinoma; PRAD, prostate adenocarcinoma; SARC, sarcoma; TGCT, testicular germ cell tumors; THCA, thyroid carcinoma; THYM, thymoma; UCEC, uterine corpus endometrial carcinoma; UCS, uterine carcinosarcoma; UVM, uveal melanoma. Source data

As outlined in Fig. 2a, the expression-based predictive models of DEPMAP dependencies were transposed using the transcriptomic profiles of 9,596 TCGA patients, following alignment to account for differences between the expression profiles of cell lines and tumor biopsies with varying stromal content. The importance of transcriptional alignment was evident from the strong correlation of the 1,966 cross-validated gene essentiality models with the tumor purity of TCGA samples (Fig. 2b). To overcome this issue, expression data from DEPMAP and TCGA were quantile normalized and transformed by contrastive principal-component analysis (cPCA), which is a generalization of the PCA that detects correlated variance components that differ between two datasets. The removal of the top four principal components (cPC1–4) between the DEPMAP and TCGA transcriptomes significantly reduced the correlation of tumor dependencies with tumor purity (Fig. 2b) and improved the alignment of the expression-based dependency models (Fig. 2c,d and Extended Data Fig. 1f–h). Enrichment analysis of gene essentiality scores with correlation coefficients that changed the most between the pre- and post-aligned models revealed a significant enrichment of pathways related to the stroma (Supplementary Table 4). Combined, these data demonstrate that without transcriptional alignment, the predicted gene essentialities in patient samples were strongly correlated with tumor purity, which should not be the case when one considers that these dependency models were generated using cultured cancer cell lines without stroma.

To further benchmark the accuracy of TCGA_DEPMAP_, we tested whether gene essentiality in patient tumors could predict tumor lineages and oncogene dependencies, as has been reported in the cell-based dependency maps^8^. The predicted negative values indicate higher predicted essentiality. Unsupervised clustering of gene essentialities across TCGA_DEPMAP_ revealed striking lineage dependencies (Fig. 2e and Supplementary Table 5), including well-known oncogenes such as *KRAS* (Fig. 2f,g) and *BRAF* (Fig. 2h,i). For example, *KRAS* essentiality was markedly stronger in *KRAS*-mutant stomach adenocarcinoma (STAD), rectal adenocarcinoma (READ), pancreatic adenocarcinoma (PAAD) and colon adenocarcinoma (COAD) lineages (Fig. 2f,g), whereas *BRAF* essentiality was strongest in *BRAF*-mutant skin cutaneous melanoma (SKCM) (Fig. 2h,i). We more broadly compared oncogene essentiality in TCGA patients with or without a gain-of-function (GOF) event (mutation or amplification), using the list of 100 cross-validated models for oncogenes from the Cosmic Cancer Gene Census (https://cancer.sanger.ac.uk/census). Of the 100 oncogenes, a total of 85 gene essentialities predicted stronger dependencies in patients with a GOF event (Supplementary Table 6). To ensure that the associations between dependencies and mutations were not due to the same underlying predictive features, the accuracy of elastic-net models to predict essentiality and somatic mutations in the same genes were compared. The comparison was restricted to genes with cross-validated models of essentiality and somatic mutations with >2% prevalence (*n* = 891 models). The elastic-net models were allowed to select the most informative predictive features for mutation and essentiality for each gene, as the best predictors for essentiality may not be the best features to predict mutation. Comparison of the area under the curve (AUC) of the two model sets revealed that transcriptomic features were significantly more predictive of gene essentiality compared to mutational status (Extended Data Fig. 1i). Considering that the expression-only models of essentiality did not include genomic features, these data further demonstrate that the essentiality scores in TCGA_DEPMAP_ can be independently correlated with genomic features in patient tumors. Combined with the evidence that cross-validated gene essentiality models accurately predict cancer lineages, these data suggest that the cross-validated gene essentiality models are accurate and interpretable across a wide range of biological contexts, including oncogenic dependencies.

### Selective dependencies in TCGA_DEPMAP_

**Extended Data Fig. 2:**
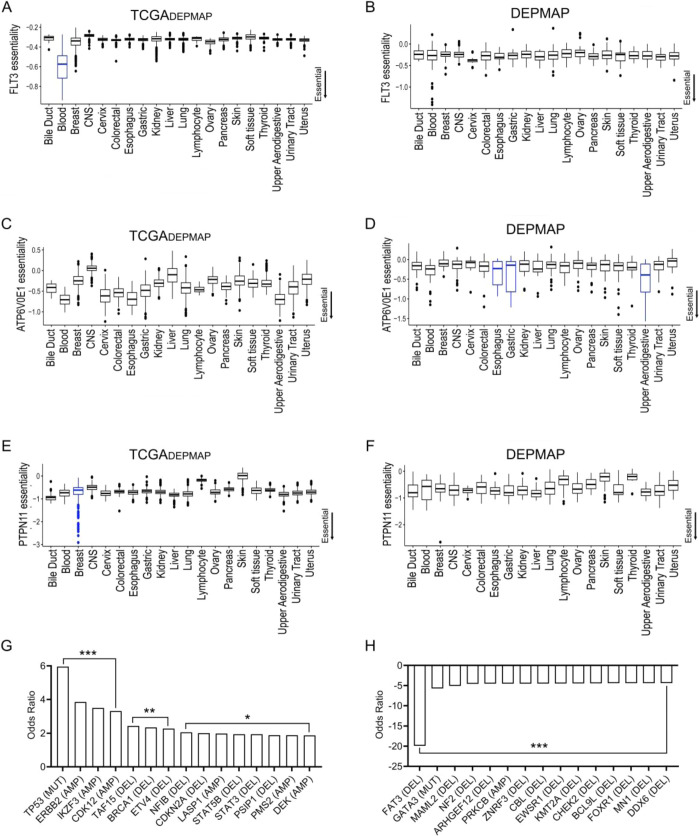
Examples of dependencies with different selectivity profiles across TCGA_DEPMAP_ and DEPMAP cohorts. (**a**) *FLT3* was classified as a strongly selective dependency (SSD) with markedly higher dependency in blood lineage cancers of TCGA_DEPMAP_ (blue bar, n = 7,021), (**b**) whereas *FLT3* showed higher dependency in some blood lineage cancers but does not meet the threshold of an SSD in DEPMAP (n = 810). (**c**) *ATPV6V0E1* essentiality scores varied widely across TCGA_DEPMAP_ (n = 7,021), (**d**) while *ATPV6V0E1* was classified as an SSD that was restricted to only a few lineages in DEPMAP (blue bars, n = 810). (**e**) *PTPN11* was classified as an SSD with very strong dependencies in a subset of breast cancer patients in TCGA_DEPMAP_ (blue bar, n = 7,021), (**f**) whereas no selectivity of *PTPN11* essentiality was detected in DEPMAP (n = 810). For (A-F), the center horizontal line represents the median (50th percentile) value. The box spans from the 25th to the 75th percentile. The whiskers indicate the 5th and 95th percentiles (**g**) Top cancer driver mutations enriched in TCGA_DEPMAP_ breast cancer patients that were highly dependent on *PTPN11*. (**h**) Top cancer driver mutations depleted in TCGA_DEPMAP_ breast cancer patients that were highly dependent on *PTPN11*. For (**g**, **h**), ***FDR < 0.01, **P < 0.01, and *P < 0.05, as determined by Fisher exact test. Source data

Strongly selective dependencies (SSDs) have been characterized in cell-based maps using the normality likelihood ratio test (NormLRT) to rank whether an essentiality fits a normal or *t*-skewed distribution (selective) across the cohort^20,29^. A strength of this approach is the ability to rank SSDs regardless of the underlying mechanisms of dependency (for example, lineage, genetic and expression). To compare the SSDs in patients with cancer and cell models, NormLRT was applied to gene effect scores for the cross-validated essentiality models in TCGA_DEPMAP_ and DEPMAP, respectively. Most SSDs (NormLRT > 100) correlated well between TCGA_DEPMAP_ and DEPMAP (*r* = 0.56, *P* < 0.0001), including *KRAS*, *BRAF*, *MYCN* and many other known SSDs (Fig. 2j and Supplementary Table 7). Although most SSDs correlated well between TCGA_DEPMAP_ and DEPMAP, there were several examples where the SSDs differed between patients and cell models (Fig. 2j,k). Notably, the druggable oncogenes (for example, *FLT3* and *PTPN11*) were more prominent SSDs in TCGA_DEPMAP_ patients than DEPMAP cell lines, whereas other notable SSDs in the DEPMAP (for example, ATP6V0E1) were less noticeable in TCGA_DEPMAP_ (Fig. 2j,k). The top predictive features for essentiality of *FLT3* (self-expression) and *ATPV6V0E1* (paralog expression) did not differ between DEPMAP and TCGA_DEPMAP_, yet the distribution and prevalence of strong dependency scores varied across lineages between patients and cell lines (Extended Data Fig. 2a–d). Likewise, the dependency on *PTPN11* (SHP2) was noticeably more selective in TCGA_DEPMAP_ than DEPMAP (Fig. 2j,k), which was reflected by greater essentiality in a subset of patients with breast cancer (BRCA) (Extended Data Fig. 2e) that was absent from BRCA cell lines (Extended Data Fig. 2f). A Fisher’s exact test of the genetic drivers that were enriched in TCGA_DEPMAP_ patients with BRCA that were most dependent on *PTPN11* included *TP53* mutations and *HER2/ERBB2* amplifications (Extended Data Fig. 2g), whereas *FAT3* deletions and *GATA3* mutations were depleted in these patients (Extended Data Fig. 2h). Particularly in the case of *HER2*, which signals through *SHP2* and the RAS pathway, these data fit with the observation that RAS pathway inhibition, including SHP2 inhibitors, are more potent in the three-dimensional (3D) versus two-dimensional (2D) context^30,31^. Thus, the presence of TCGA_DEPMAP_ patients with BRCA that were highly dependent on *PTPN11* is likely due to the 3D context of patient tumors, whereas DEPMAP BRCA cell lines with similar genetic drivers are not *PTPN11* dependent due to the 2D context of cultured cells. Collectively, these data demonstrate that identifying SSDs can be impacted by different prevalence and distributions of the underlying drivers in patients and cell models, which can be overcome by patient-relevant dependency maps, such as TCGA_DEPMAP_.

### Clinical phenotypes and outcomes in TCGA_DEPMAP_

**Fig. 3:**
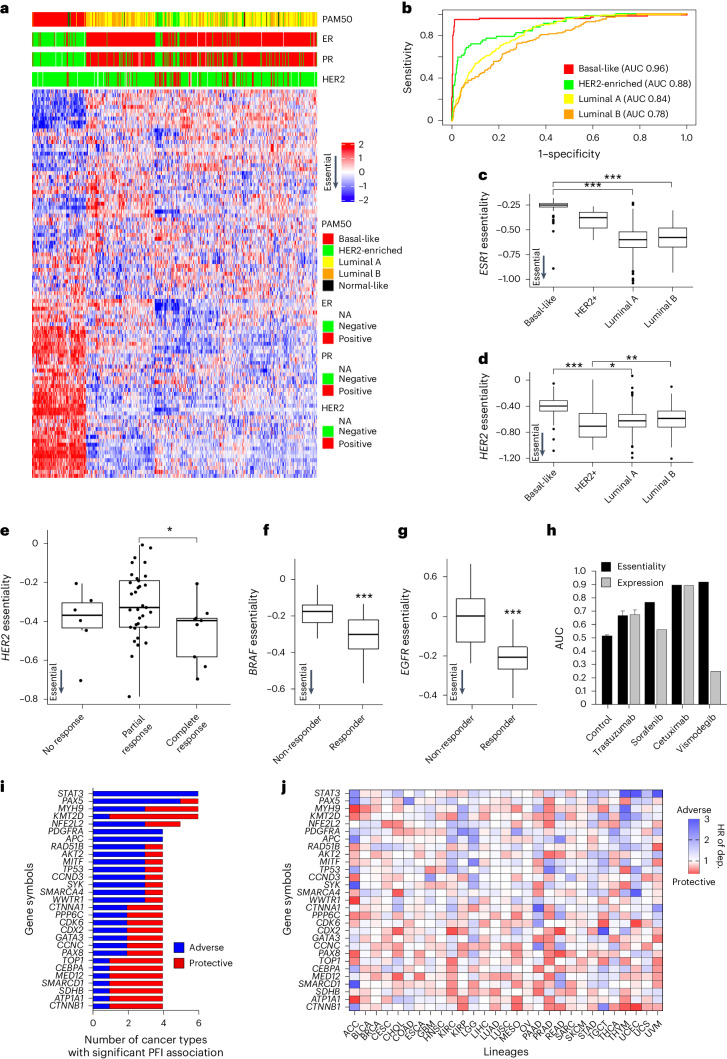
Translating TCGA_DEPMAP_ to clinically relevant phenotypes and outcomes. **a**, Unsupervised clustering of the top 100 dependencies in TCGA breast cancer patients. **b**, A ROC–AUC analysis was used to test the accuracy of calling breast cancer subtypes using the top 100 dependencies. **c**, *ESR1* dependencies are strongest in ER-positive luminal BRCA (*n* = 96 for basal-like, *n* = 57 for HER2^+^, *n* = 231 for luminal A, *n* = 126 for luminal B and *n* = 7 for normal-like). **d**, *HER2* dependencies are strongest in *HER2*-amplified BRCA (*n* = 96 for basal-like, *n* = 57 for HER2^+^, *n* = 231 for luminal A, *n* = 126 for luminal B and *n* = 7 for normal-like) **e**, *HER2* dependency predicts trastuzumab response in patients with BRCA (*n* = 6 for no response, *n* = 33 for partial response and *n* = 9 for complete response). **f**, *BRAF* dependency predicts sorafenib response in patients with hepatocellular cancer (*n* = 46 for non-responder and *n* = 21 for responder). **g**, *EGFR* dependency predicts cetuximab response in patients with head and neck cancer (*n* = 26 for non-responder and *n* = 14 for responder). For **c**–**g**, *P < 0.05, **P < 0.01 and ***P < 0.001, as determined by the Wilcoxon rank-sum test for two-group comparison and Kruskal–Wallis test followed by a Wilcoxon rank-sum test with multiple test correction for the multi-group comparison. For boxplots in **c**–**g**, the center horizontal line represents the median (50th percentile) value. The box spans from the 25th to the 75th percentile. The whiskers indicate the 5th and 95th percentiles. **h**, AUC values for drug response predictions based on essentiality, expression and random essentiality scores generated via random sampling (control). **i**, Top gene essentialities associated with the PFI by univariate Cox proportional hazard regression model across multiple lineages in TCGA_DEPMAP_ (Benjamini–Hochberg, FDR < 0.2). **j**, HRs of the top essentialities across TCGA_DEPMAP_. Blue indicates a greater dependency associated with worse outcome and red indicates a greater dependency is associated with better outcome. *P* values and HRs are shown in Supplementary Table 9. Source data

Another strength of translational tumor dependency maps is the ability to assess the impact of gene essentiality on clinically relevant phenotypes, such as molecular subtyping, therapeutic response and patient outcomes. To evaluate the utility of TCGA_DEPMAP_ for therapy-relevant patient stratification, an unsupervised clustering of the 100 most variable gene dependencies was performed using the TCGA_DEPMAP_ BRCA cohort (Fig. 3a). The 100-dependency signature (DEP100) performed comparably to the established PAM50 signature^32^ in classifying BRCA subtypes (AUC > 0.8 for most subtypes), despite only three overlapping genes between PAM50 and DEP100 (Fig. 3b). Dependency subtyping with DEP100 predicted significantly higher *ESR1* essentiality in ER-positive tumors (Fig. 3c) and higher *HER2* essentiality in *HER2*-amplified tumors (Fig. 3d). Finally, due to the limited accessibility of therapeutic response data in TCGA^33^, we identified nine clinical datasets for molecular therapeutics of tumor dependencies for which we had accurate models and sufficient statistical power^34–36^. Of these nine datasets, we found seven out of nine dependency models significantly predicted clinical responses and performed better or comparable to the target gene expression in predicting therapeutic responses (Fig. 3e–h and Supplementary Table 8). Of the two nonsignificant datasets, both trended in the correct direction and would likely reach statistical significance with larger cohort sizes. Taken together, these data establish the physiological relevance of TCGA_DEPMAP_ to associate dependencies with common clinicopathological features, such as molecular subtyping and therapeutic response.

The ability to associate gene essentiality with patient survival is a unique strength of TCGA_DEPMAP_, which is not accessible using cell-based dependency maps. Moreover, outcomes driven by perturbations of oncogenic pathways and genetic drivers of human cancers are likely not captured by gene expression alone and rather require a readout of gene essentiality. To test this possibility, the cross-validated gene essentiality models (*n* = 1,966) were tested for association with the progression-free interval (PFI) in TCGA_DEPMAP_. Among 29 cancer lineages that are well powered for PFI analysis^33^, 105 known genetic drivers of human cancer were significantly associated with the PFI of TCGA patients (Supplementary Table 9), including 29 that were prognostic in at least four cancer lineages (Fig. 3i,j). For example, a stronger dependency on the druggable oncogene, *STAT3* (ref. ^35^), was significantly associated with a shortened time to disease progression of six different cancers (Fig. 3i,j). Likewise, multiple other prevalent genetic drivers of human malignancies were associated with a significantly shorter PFI, including *PAX5* and *PDGFRA* (Fig. 3i,j). Both proteins have been investigated previously as prognostic indicators of poor outcomes by expression analysis in patient biopsies^37,38^ and this study shows that dependency on these oncogenes is associated with worse outcome in patients using a translational dependency map.

### Synthetic lethalities in TCGA_DEPMAP_

**Extended Data Fig. 3:**
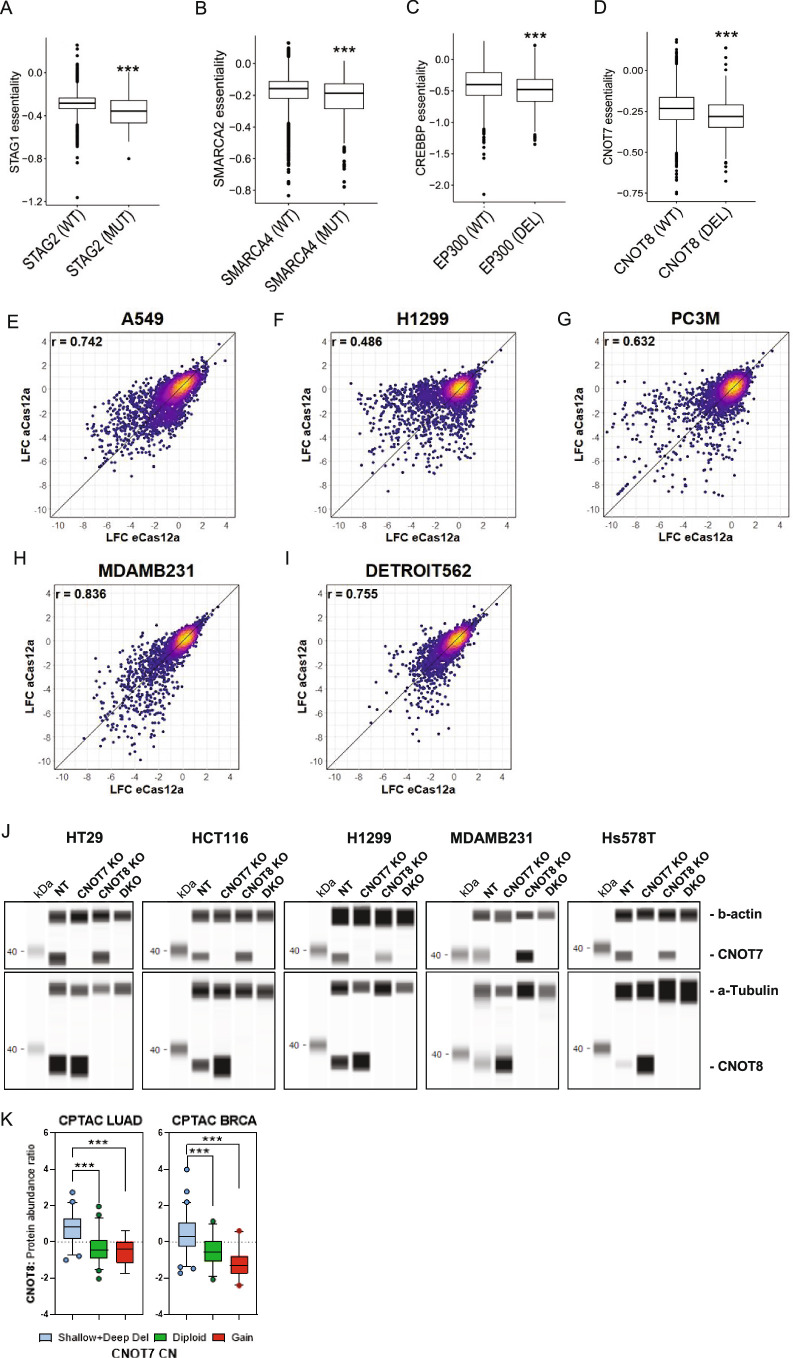
Characterization of synthetic lethalities. (**a**) *STAG1* synthetic lethality with *STAG2* mutation (n = 163 for *STAG2*^MUT^ and n = 7,418 for *STAG2*^WT^), (**b**) *SMARCA2* synthetic lethality with *SMARCA4* mutation (n = 223 for *SMARCA4*^MUT^ and n = 7,358 for *SMARCA4*^WT^), (**c**) *CREBBP* synthetic lethality with *EP300* mutation (n = 937 for *EP300*^DEL^ and n = 6,644 for *EP300*^WT^), and (**d**) *CNOT7* synthetic lethality with *CNOT8* deletion are examples of synthetic lethalities that were detected by TCGA_DEPMAP_. (n = 550 for *CNOT8*^DEL^ and n = 7,031 for *SMARCA4*^WT^) ***P < 0.001, as determined by the Wilcoxon rank-sum test. (E-I) Comparison of multiplexed CRISPR/Cas12 screens performed using AsCas12a and EnAsCas12a enzymes. Analysis was performed using a Pearson’s correlation and coefficients (*r*) are displayed on the graphs. (**j**) Simple Western blots of protein expression of *CNOT7, CNOT8* and housekeeping control Beta-Actin of nontargeting (NT) control, single (KO) and dual (DKO) knockout cells 3 days after CRISPR/RNP electroporation. (**k**) Plots showing the protein abundance ratio of *CNOT8* (Y-axis) and copy number status of *CNOT7* (X-axis) in the CPTAC Lung Adenocarcinoma (LUAD) and Breast Cancer (BRCA) cohorts showing a significant upregulation of *CNOT8* protein in tumors with *CNOT7* copy number loss (shallow and deep deletions) compared to diploid and gain tumors (for LUAD n = 7 for gain, n = 51 for diploid and n = 55 for shallow deletion; for BRCA n = 22 for gain, n = 33 for diploid and n = 67 for shallow deletion). For (A-D and K), the center horizontal line represents the median (50th percentile) value. The box spans from the 25th to the 75th percentile. The whiskers indicate the 5th and 95th percentiles. The two-sided Wilcoxon rank test was used for (A-D) ***p < 0.001 and ***p < 0.001 as determined by Student’s unpaired, two-tailed t-test for (K). Source data

In addition to illuminating lineage and oncogenic dependencies, the DEPMAP has dramatically expanded the list of potential synthetic lethalities (the loss of a gene sensitizes tumor cells to inhibition of a functionally redundant gene within the same pathway)^6,16,17,39,40^; however, one of the current limitations of the DEPMAP is that the available cancer cell models do not yet fully recapitulate the genetic and molecular diversity of TCGA patients^25^. Thus, we assessed the landscape of predicted synthetic lethalities with loss-of-function (LOF) events (damaging mutations or deletions) in TCGA_DEPMAP_. Lasso regression analysis of gene essentiality profiles and 25,026 LOF events detected in TCGA_DEPMAP_ yielded 633,232 synthetic lethal candidates (FDR < 0.01) (all candidates added as an R object to a figshare repository), which were too numerous to experimentally validate by current methods. To prioritize the synthetic lethal candidates, the gene interaction scores were correlated with the mutual exclusivity of corresponding mutations in TCGA_DEPMAP_, which narrowed the list to 28,609 candidates (FDR < 0.01). Multiple additional criteria were then applied to refine the list further by enriching for predicted paralogs with close phylogenic distance to prioritize candidates with redundant functions due to sequence homology. All told, this approach identified many known synthetic lethal pairs (for example, *STAG1*/STAG*2*, *SMARCA2*/*SMARCA4* and *EP300*/*CREBBP*)^41–43^ and previously untested synthetic lethal candidates, demonstrating that TCGA_DEPMAP_ is well powered to predict synthetic lethal relationships with LOF events in patient tumor biopsies (Extended Data Fig. 3a–d and Supplementary Table 10).

**Fig. 4:**
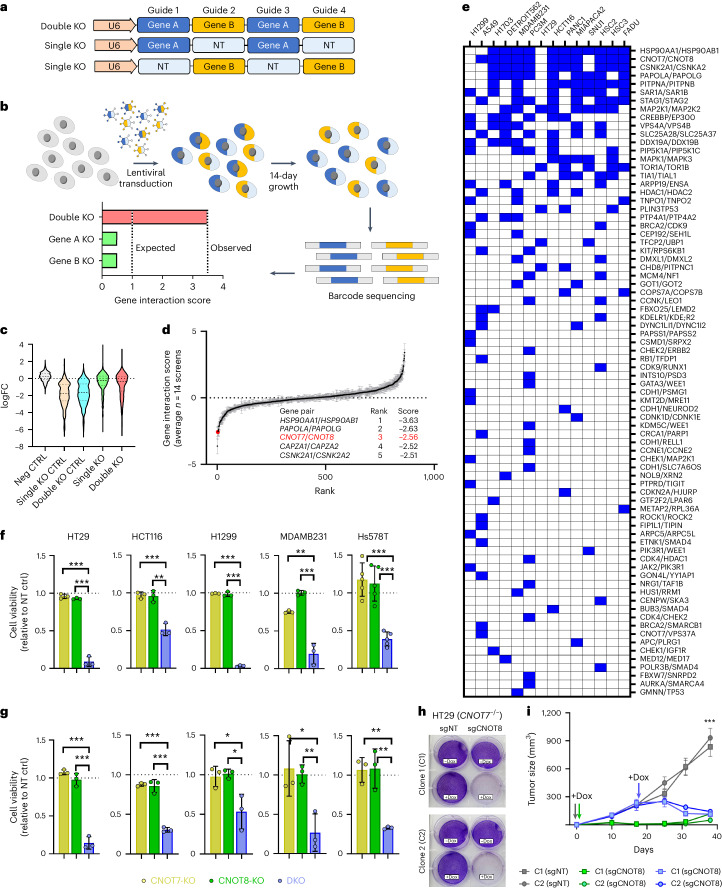
Using TCGA_DEPMAP_ to translate synthetic lethalities in human cancer. **a**, Schematic of the CRISPR/Cas12 library multiplexed guide arrays targeting one or two genes per array. **b**, Schematic of the synthetic lethality screening approach using the CRISPR/Cas12 library. All CRISPR screens were performed as *n* = 3 biological replicates per cell line. **c**, Violin plots of target-level CRISPR of the average log_2_ fold change (FC) across all tested cell lines for nontargeting (NT) guide (neg CTRL), single knockout guides targeting essential genes (single KO CTRL), DKO guides targeting essential genes (DKO CTRL), single knockout guides of TCGA_DEPMAP_ candidates (single KO) and DKO guides of TCGA_DEPMAP_ candidates (DKO). **d**, Rank plot of target-level gene interaction (GI) scores averaged across *n* = 14 cell lines in the CRISPR/Cas12 multiplexed screening (A549, DETROIT562, FADU, H1299, H1703, HCT116, HSC2, HSC3, HT29, MDAMB231, MIAPACA2, PANC1, PC3M and SNU1), including the top five synthetic lethalities (table insert). The black line indicates the mean and gray error bars show ±s.e.m. **e**, Distribution of synthetic lethal candidates from TCGA_DEPMAP_ with experimental evidence of synthetic lethality in the CRISPR/Cas12 multiplexed screening across 14 cancer cell lines. A blue box indicates a GI score < −2. **f**,**g**, Cell viability assessed by CellTiterGlo (CTG) luminescence at 7 days after single (KO) or dual (DKO) *CNOT7*/*CNOT8* knockouts, normalized to NT controls in five cell lines grown in 2D monolayers (**f**) or 3D spheroids (**g**); *n* = 3 biological replicates per cell model per condition with the exception of *n* = 5 biological replicates for Hs578T grown in 2D monolayer. Error bars are mean ± s.d. **h**, Crystal violet staining of *CNOT7*^−/−^ clones C1 and C2 stably expressing nontargeting (sgNT) or *CNOT8*-targeting (sgCNOT8) dox-inducible guide constructs, following 7 days of dox treatment (Methods). **i**, Tumor xenograft studies of HT29 clones grown in mice fed dox-containing food from day 0 (gray and green lines) or beginning on day 19 (blue lines). *n* = 5 mice per group. Error bars are ±s.d. Asterisks in **f**, **g** and **i** reflect two-tailed, unpaired Student’s *t*-test *P* values; *P < 0.05; **P < 0.01; ***P < 0.001. Source data

Synthetic lethalities that were predicted with LOF events in the TCGA_DEPMAP_ (*n* = 604 pairs) were experimentally tested using a multiplexed CRISPR/AsCas12a screening approach across representative cell models of five cancer lineages (Fig. 4a,b). Additional pairs (*n* = 261 controls) were added to the library to control for screen performance, including essential paralog pairs and nonessential pairs of tumor suppressor genes (TSGs) and interacting partners (Supplementary Table 10). An initial pilot screen was performed using five cancer cell models, which experimentally validated 69 TCGA_DEPMAP_ synthetic lethalities in at least one representative cell model (Supplementary Table 11). As these data were being generated, an enhanced AsCas12a (enAsCas12a) enzyme was reported to be compatible with CRISPR/AsCas12a libraries^44^, enabling replication of the initial pilot screens and expansion to a total of 16 cancer cell models. Notably, the replication of the initial screens was highly concordant across the five cell models in common (average *r* = 0.69) (Extended Data Fig. 3e–i), as well as detection of increased depletion of essential controls and synthetic lethal partners compared to nonessential controls (Fig. 4c). In addition to novel pairs, multiple previously reported synthetic lethalities (*HSP90AA1*/*HSP90AB1* (ref. ^45^), *DDX19A*/*DDX19B*^45^, *HDAC1*/*HDAC2* (refs. ^45,46^), *SMARCA2*/*SMARCA4* (refs. ^45,46^), *EP300*/*CREBBP*^43^, *STAG1*/*STAG2* (refs. ^42,46^) and *CNOT7*/*8* (ref. ^47^)) were replicated across multiple cell lines in both cohorts (Supplementary Table 11), demonstrating the robustness of the multiplex CRISPR/Cas12a screening platform to test synthetic lethalities. Notably, as observed elsewhere^39,41,46^, the sensitivity to synthetic lethalities varied between cell models and lineages, implicating the prevalence of unknown modifiers of synthetic lethality that manifest in different cellular contexts and are yet to be fully understood.

Of the 604 synthetic lethalities predicted by TCGA_DEPMAP_, a total of 78 (13%) were experimentally validated in at least one representative cell model (Fig. 4d,e and Supplementary Table 11). For example, double knockout (DKO) of *CNOT7/8* was synthetic lethal in 11 out of 14 cell lines that were screened (Fig. 4e) and was orthogonally validated in five cell models by DKO using ribonucleoprotein (RNP) in both 2D monolayer and 3D spheroid assays (Fig. 4f,g). Likewise, doxycycline (dox)-inducible loss of *CNOT8* was synthetic lethal in HT29 cells that lacked *CNOT7* in both in vitro 2D monolayers (Fig. 4h) and in vivo mouse xenograft studies (Fig. 4i). Notably, loss of *CNOT7* in single knockout (KO) cells coincided with elevated CNOT8 protein (Extended Data Fig. 3j), fitting with previous observations that loss of *CNOT7* increases integration of *CNOT8* into the CCR4–NOT complex^48^. Likewise, CNOT8 protein levels were inversely correlated with *CNOT7* copy numbers in patients with lung adenocarcinoma (LUAD) and BRCA in the NCI Clinical Proteomic Tumor Analysis Consortium cohort (Extended Data Fig. 3k). Collectively, these observations demonstrate the power of TCGA_DEPMAP_ to detect patient-relevant synthetic lethal mechanisms, which can be orthogonally validated and provide therapeutic targets for drug discovery.

**Fig. 5:**
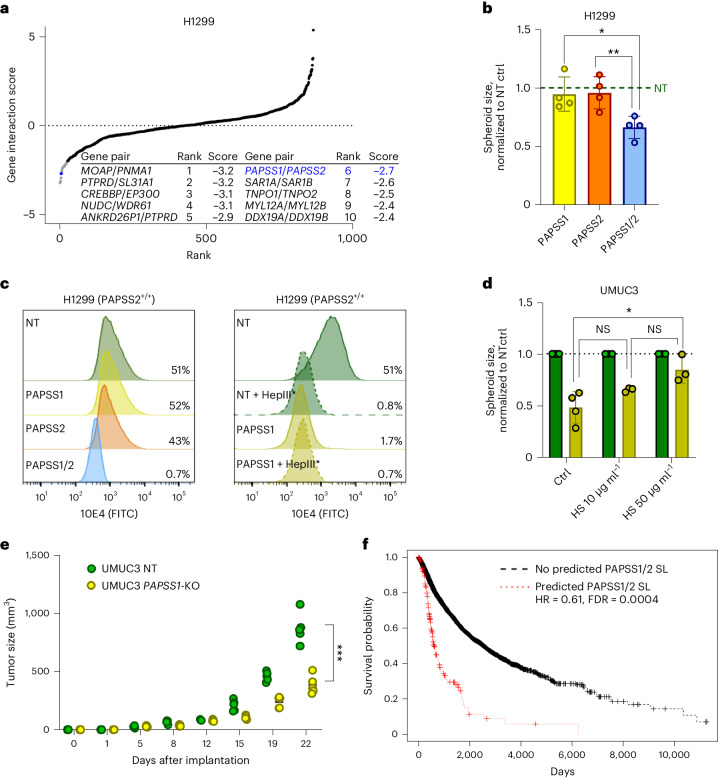
*PAPSS1* and *PAPSS2* are novel synthetic lethal paralogs detected by TCGA_DEPMAP_. **a**, Rank plot of target-level GI scores in H1299 cells, including the top ten synthetic lethalities (table insert). The novel synthetic lethality, *PAPSS1*/*PAPSS2*, is highlighted in blue. All CRISPR screens were performed as *n* = 3 biological replicates per cell line. **b**, Spheroid size of H1299 cells with single or dual *PAPSS1* and *PAPSS2* knockouts, normalized to NT control spheroids; *n* = 4 biological replicates per condition. Data show mean ± s.d. *P < 0.05 and **P < 0.01 as per unpaired, two-tailed *t*-test. **c**, Flow cytometry histogram overlay plots of viable H1299 and UMUC3 cells (DAPI^−^) showing expression of cell surface sulfonated HSPGs as measured by antibody clone 10E4-FITC. Dual loss of *PAPSS1/PAPSS2* leads to total loss of sulfonation comparable to heparinase III treatment (HepIII*) which specifically cleaves sulfonated HS chains. **d**, Growth defects of UMUC3 spheroids following deletion of *PAPSS1* (yellow bars) were partially rescued by the addition of 10 μg ml^−1^ and 50 μg ml^−1^ of exogenous HS as compared to NT control spheroids (green bars); *n* = 4 biological replicates for the untreated control and *n* = 3 biological replicates per treated condition. Data are mean ± s.d. *P < 0.05 as per unpaired, two-tailed *t*-test. **e**, Diagram showing tumor volumes over time (d, days) after in vivo implantation of 1 × 10^6^ UMUC3 NT or *PAPSS1*-KO cells in SCID/beige mice. Each dot represents an individual mouse (*n* = 5 mice per condition); ***P < 0.001, as determined by unpaired, two-tailed *t*-test of the final data point. **f**, Kaplan–Meier plot of TCGA_DEPMAP_ patients with a predicted *PAPSS1*/*PAPSS2* synthetic lethality has a worse outcome compared to the rest of the cohort, as determined by a Cox log-rank test. DAPI, 4,6-diamidino-2-phenylindole. Source data

**Extended Data Fig. 4:**
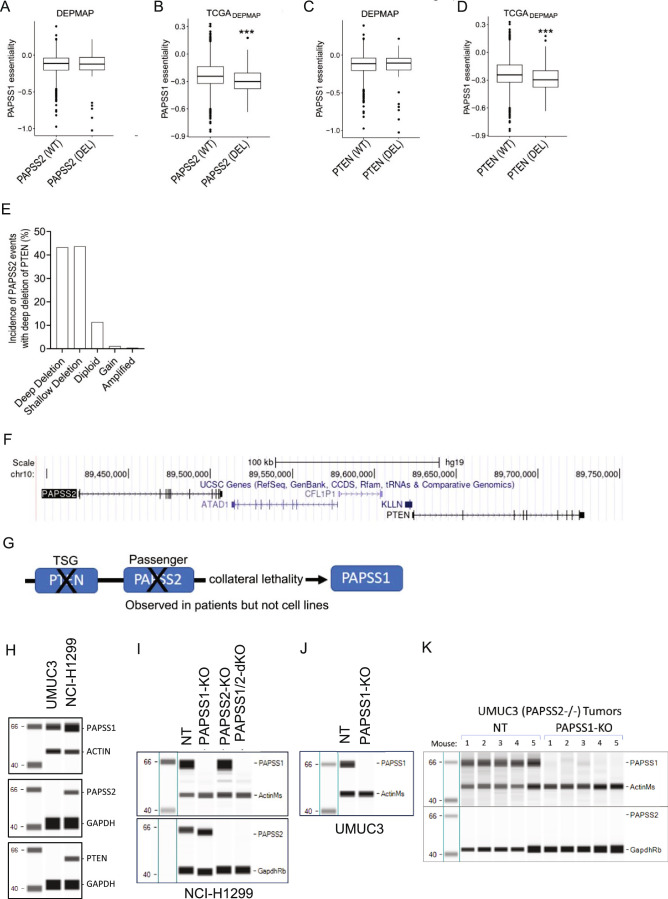
Supporting evidence of *PAPSS1/2* synthetic lethality*.* **a**, **b**) *PAPSS1* is a novel synthetic lethality in the context of *PAPSS2* deletion, which is not detectable in (**a**) DEPMAP cell lines (n = 905) and is only detectable in (**b**) TCGA_DEPMAP_ patient samples (n = 7,581). (**c**, **d**) Likewise, *PAPSS1* is not synthetic lethal with *PTEN* deletion in DEPMAP cell lines (**c**, n = 905) and is only detectable in TCGA_DEPMAP_ patient samples (**d**, n = 7,581). For (A-D), the center horizontal line represents the median (50th percentile) value. The box spans from the 25th to the 75th percentile. The whiskers indicate the 5th and 95th percentiles. (**e**) Unlike cultured cell models, *PAPSS2* is frequently co-deleted with *PTEN* in TCGA patients. (**f**) *PAPSS2* is a closely neighboring gene of *PTEN*. (**g**) A schematic representation summarizing the hypothesized synthetic lethality of *PAPSS1* that is driven by collateral deletion of *PAPSS2* with the tumor suppressor gene (TSG), *PTEN*, in patients but not cell lines. ***P < 0.001, as determined by the Wilcoxon rank-sum test. (**h**) Endogenous expression by Simple Western of PAPSS1, PAPSS2, and PTEN in the model cell lines UMUC3 and NCI-H1299. (**i**,**j**) Validation of *PAPSS1* and *PAPSS2* single (KO) and double (dKO) knockouts by RNP in spheroid experiments for NCI-H1299 (**i**) and UMUC3 (**j**). (**k**) Validation of PAPSS1 knockout in the UMUC3 xenograft experiment tumors (n = 5 tumors per condition from n = 1 independent experiment). Molecular weight marker lanes are shown in kDa. Data shown in (**h-j**) are representative from at least 3 independent experiments. The two-sided Wilcoxon rank test was used for (A-D), ***P < 0.001. Source data

Another discovery using TCGA_DEPMAP_ was the prediction of *PAPSS1* synthetic lethality with deletion of *PAPSS2* and the neighboring tumor suppressor, *PTEN*, which were frequently co-deleted in TCGA patient tumors (43% co-incidence) yet were largely unaffected in cancer cell lines (Extended Data Fig. 4a–g). *PAPSS1*/*PAPSS2* are functionally redundant enzymes essential for synthesis of 3′-phosphoadenosine 5′-phosphosulfate (PAPS), which is required for all sulfonation reactions^49^, suggesting that loss of *PAPSS1*/*PAPSS2* is synthetic lethal due to the inability to sulfonate proteins. To test this hypothesis, *PAPSS1*/*PAPSS2* were targeted in H1299 spheroids by RNP, followed by measurement of spheroid growth and sulfonation levels of heparan sulfate (HS) proteoglycan (HSPG) chains on the cell surface by flow cytometry. Confirming the CRISPR/Cas12 screen data (Fig. 5a), dual loss of *PAPSS1* and *PAPSS2* significantly reduced H1299 spheroid growth compared to controls (Fig. 5b and Extended Data Fig. 4h,i), which coincided with loss of HSPG sulfonation (Fig. 5c). Likewise, targeting *PAPSS1* by RNP in UMUC3 cells, which endogenously lack *PAPSS2* and *PTEN*, also significantly depleted HSPG sulfonation and coincided with significant spheroid growth reduction, which could be rescued by addition of exogenous heparan sulfate (Fig. 5d and Extended Data Fig. 4h,j). Finally, *PAPSS1*/*PAPSS2* synthetic lethality was confirmed in vivo, as demonstrated by a significant tumor growth reduction of UMUC3 tumors without *PAPSS1* and *PAPSS2* compared to control tumors lacking only *PAPSS2* (Fig. 5e and Extended Data Fig. 4k). Taken together, these data demonstrate that translational dependency maps, such as the TCGA_DEPMAP_ are powerful tools to uncover previously underrepresented synthetic interactions in cancer models that are likely to be patient relevant.

TCGA_DEPMAP_ is unique in its ability to uncover potential synthetic lethalities that can be related to patient outcomes, enabling the prioritization of the experimentally validated synthetic lethalities that correlate with the worst outcome and therefore likely to have the greatest clinical impact if druggable. To test this possibility, a Cox log-rank test was used to assess overall survival (OS) of TCGA patients who correlated with predicted gene essentiality by TCGA_DEPMAP_ and LOF events (mutation, deletion or both) of the putative synthetic lethal partner. After controlling for tumor lineage, *PAPSS1* dependency in TCGA_DEPMAP_ was correlated with significantly worse OS (hazard ratio (HR) = 0.61, *P* = 0.0004) in patients with *PAPSS2* deletion (Fig. 5f), demonstrating that *PAPSS1* is a synthetic lethality target with potentially high translational impact. Collectively, these data demonstrate that translational dependency maps can enable the discovery, validation and translation of synthetic lethalities.

### Constructing PDXE_DEPMAP_

**Fig. 6:**
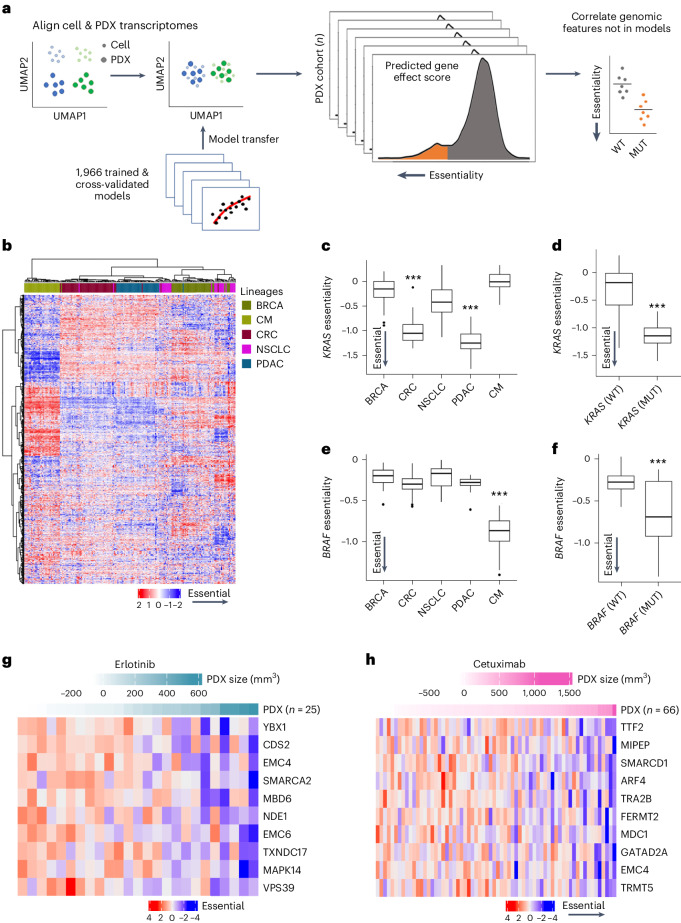
Building a translational dependency map in patient-derived xenografts: PDXE_DEPMAP_. **a**, Schematic of gene essentiality model transposition from DEPMAP to PDXE, following alignment of genome-wide expression data to account for differences in homogeneous cultured cell lines and PDX samples with contaminating stroma. **b**, Unsupervised clustering of predicted gene essentiality scores across five lineages in PDXE_DEPMAP_ confirmed similar lineage drivers of gene dependencies, as observed in TCGA_DEPMAP_. Blue indicates genes with stronger essentiality and red indicates genes with less essentiality. **c**, *KRAS* dependency was enriched in PDXE_DEPMAP_ lineages with high frequency of *KRAS* GOF mutations, including CRC and PDAC. *n* = 43 for BRCA, *n* = 51 for CRC, *n* = 27 for NSCLC, *n* = 39 for PDAC and *n* = 32 for CM. **d**, *KRAS* essentiality correlated with *KRAS* mutations in all PDXE_DEPMAP_ lineages (*n* = 74 for *KRAS*^mut^ and *n* = 117 for *KRAS*^wt^). **e**, *BRAF* dependency in PDXE_DEPMAP_ was enriched in CM, which has a high frequency of GOF mutations in *BRAF*. *n* = 43 for BRCA, *n* = 51 for CRC, *n* = 27 for NSCLC, *n* = 39 for PDAC and *n* = 32 for CM. **f**, *BRAF* essentiality correlated with *BRAF* mutations in all TCGA_DEPMAP_ lineages (*n* = 32 for *BRAF*^mut^ and *n* = 159 for *BRAF*^wt^). For **c**–**f**, the center horizontal line represents the median (50th percentile) value. The box spans from the 25th to the 75th percentile. The whiskers indicate the fifth and 95th percentiles. **g**, Top correlated gene essentiality models that correlate with PDX response to erlotinib in PDXE_DEPMAP_. **h**, Top correlated gene essentiality models that correlate with PDX response to cetuximab in PDXE_DEPMAP_. ***P < 0.001, as determined by the Wilcoxon rank-sum test for two-group comparison (**d** and **f**) and Kruskal–Wallis test followed by a Wilcoxon rank-sum test with multiple test correction for a multi-group comparison (**c** and **e**). NSCLC, non-small cell lung cancer. Source data

In addition to building TCGA_DEPMAP_, a similar approach was applied to generating an orthogonal translational dependency map using the PDX Encyclopedia (PDXE_DEPMAP_)^50^. As outlined in Fig. 6a, PDXE_DEPMAP_ was assembled by transferring the cross-validated 1,966 expression-only models from the DEPMAP to the PDXE (*n* = 191 tumors) using the aligned genome-wide expression profiles from the PDXE (Supplementary Table 12). Unsupervised clustering of gene essentialities across five well-represented lineages in PDXE_DEPMAP_ confirmed that lineage is a key driver of gene dependencies (Fig. 6b), fitting with the observations made in TCGA_DEPMAP_ (Fig. 2e). PDXE_DEPMAP_ also detected markedly stronger *KRAS* essentiality in *KRAS*-mutant PDX of pancreatic ductal carcinoma (PDAC) and colorectal carcinoma (CRC) lineages (Fig. 6c,d), whereas *BRAF* essentiality was strongest in *BRAF*-mutant PDX of cutaneous melanoma (CM) (Fig. 6e,f). These data collectively demonstrate that the PDXE_DEPMAP_ performed comparably to TCGA_DEPMAP_ and is well powered to detect gene essentiality signals in PDX models.

In addition to orthogonal validation of TCGA_DEPMAP_, a unique strength of PDXE_DEPMAP_ is the ability to assess gene essentiality in the context of therapeutic responses across five cancer lineages and 15 molecular therapies^50^. To test the ability of gene essentiality to predict the response to corresponding targeted therapies, the change in PDX burden from baseline to experimental end point was correlated with target gene essentiality. This revealed that 80% of drugs (12 of 15) were significantly correlated (*P* < 0.05) with the predicted essentiality of the target gene (Supplementary Table 13). For example, trastuzumab response in the PDXE_DEPMAP_ was strongly predicted by *HER2* dependency (*R* = 0.4849, *P* = 0.002, AUC = 0.75), in line with the predictive power of *HER2* dependency on trastuzumab responsiveness in patients with *HER2*-amplified BRCA (Fig. 3e). Other examples, such as erlotinib (*R* = 0.4937, *P* = 0.01, AUC = 0.78) and cetuximab (*R* = 0.2293, *P* = 0.06, AUC = 0.83), which target the same gene (*EGFR*), provide the opportunity to explore dependency mechanisms of therapeutic resistance across modalities. Comparisons of PDX responses to erlotinib or cetuximab revealed dependencies within two common pathways: the SWI/SNF complex (*SMARCA2* and *SMARCD1*) and protein trafficking (*EMC4*, *EMC6*, *VPS39* and *MAPK14*) (Fig. 6g,h). Notably, components of both pathways have been implicated in resistance to *EGFR* inhibitors^51,52^, suggesting that targeting these dependencies would likely improve patient outcomes. Taken together, these data demonstrate the ability of gene essentiality to predict therapeutic response and highlight the translatability of PDX modeling to patient-relevant clinical outcomes.

### Translating gene tolerability in GTEX_DEPMAP_

**Fig. 7:**
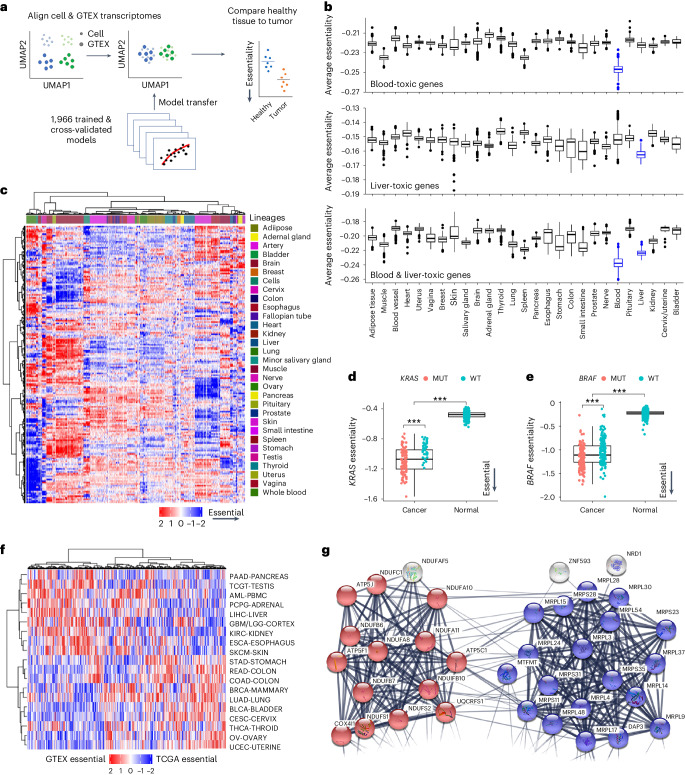
Building a translational dependency map in normal tissues: GTEX_DEPMAP_. **a**, Schematic of gene essentiality model transposition from DEPMAP to GTEX, following alignment of genome-wide expression data to account for differences in homogeneous cultured cell lines and healthy tissue biopsies. **b**, Average gene essentiality profile across healthy tissues of GTEX_DEPMAP_ (*n* = 17,382) for molecular targets with known liver and blood toxicities (in blue). **c**, Unsupervised clustering of predicted gene essentiality scores across healthy tissues. Blue indicates genes with stronger essentiality and red indicates genes with less essentiality. **d**, *KRAS* essentiality is significantly higher in PAAD with GOF mutations compared to healthy pancreas in GTEX_DEPMAP_ (*n* = 146 for cancer with *n* = 106 *KRAS*^mut^ and *n* = 40 *KRAS*^wt^, *n* = 328 for normal) **e**, *BRAF* essentiality is significantly higher in SKCM with GOF mutations compared to normal skin GTEX_DEPMAP_ (*n* = 319 for cancer with *n* = 165 *BRAF*^mut^ and *n* = 154 *BRAF*^wt^, *n* = 1,809 for normal) For **b**, **d**, and **e**, the center horizontal line represents the median (50th percentile) value. The box spans from the 25th to the 75th percentile. The whiskers indicate the fifth and 95th percentiles. **f**, Global differences between the predicted target efficacy score (TCGA_DEPMAP_) and the healthy tissue-of-origin tolerability score (GTEX_DEPMAP_). **g**, STRING network analysis of the top 100 LUAD targets with the greatest predicted tolerability in healthy lung reveals significant connectivity (*P* < 1 × 10^−16^) and gene ontology enrichment oxidative phosphorylation (blue-colored spheres; *P* = 5.8 × 10^−11^) and mitochondrial translation (red-colored spheres; *P* = 2.9 × 10^−20^). ***P < 0.001, as determined by a Wilcoxon rank-sum test for two-group comparison and Kruskal–Wallis test followed by a Wilcoxon rank-sum test with multiple test correction for a multi-group comparison (**d** and **e**). Source data

A final objective of this study was to define gene essentiality in the context of healthy tissues, which would provide a resource for prioritizing tumor dependencies with the best predicted tolerability. To achieve this objective, the expression-based dependency models from DEPMAP were transposed using the aligned expression data from GTEX (GTEX_DEPMAP_), a compendium of deeply phenotyped normal tissues collected from postmortem healthy donors (*n* = 948)^28^ (Fig. 7a and Supplementary Table 14). To assess the sensitivity of GTEX_DEPMAP_ to dependencies with low tolerability, the molecular targets of drugs with reported toxicities in the liver and blood (*n* = 241) were compared across GTEX_DEPMAP_ (Supplementary Table 15). This revealed that the average essentiality was higher in liver and blood than other normal tissues (Fig. 7b). Likewise, unsupervised clustering of the 1,966 cross-validated gene essentiality models revealed strong tissue-of-origin dependencies in healthy organs (Fig. 7c), suggesting that tissue-specific biological context also contributes to gene essentiality in normal physiological settings. Taken together, these data demonstrate that GTEX_DEPMAP_ is sensitive to known toxicities, which cluster around different healthy organ types.

**Extended Data Fig. 5:**
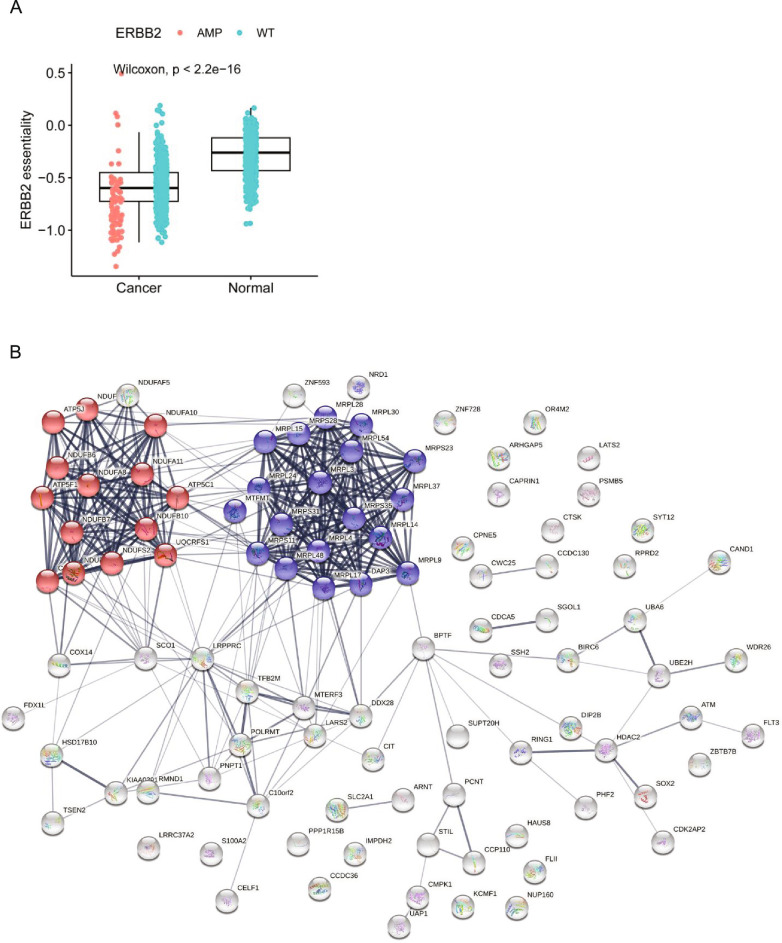
Essentiality profiles of genes in cancer versus normal tissues. (**a**) *ERBB2* essentiality is significantly higher in malignant breast cancer with *ERBB2* amplifications (TCGA_DEPMAP_, n = 137 for *ERBB2*^AMP^ and n = 932 for *ERBB2*^WT^) compared with normal breast (GTEX_DEPMAP_, n = 459). ***P < 0.001, as determined by the Wilcoxon rank-sum test. For the boxplot, the center horizontal line represents the median (50th percentile) value. The box spans from the 25th to the 75th percentile. The whiskers indicate the 5th and 95th percentiles. (**b**) STRING network analysis of the top 100 LUAD targets with the greatest predicted tolerability in normal lung reveals significant connectivity (p < 1 × 10^−16^) and gene ontology enrichment for oxidative phosphorylation (blue colored spheres; p = 5.8 × 10^−11^) and mitochondrial translation (red-colored spheres; p = 2.9 × 10^−20^). Source data

Comparing essentiality scores of known druggable oncogenes in TCGA_DEPMAP_ with GTEX_DEPMAP_ revealed greater dependency in malignant tissues versus a healthy tissue of origin. For example, *KRAS* and *BRAF* essentialities seem to be concomitantly dependent on lineage and genetic drivers, as the healthy tissues of origin were predicted to be significantly less affected in the GTEX_DEPMAP_ compared to TCGA_DEPMAP_ (Fig. 7d,e). Likewise, similar observations were made for other oncogenic drivers that are approved therapeutic targets in patients with cancer, such as *HER2*-amplified BRCA (Extended Data Fig. 5a). In contrast, there was markedly less separation in the predicted essentialities of malignant tumors and healthy tissues of origin for molecular therapies that have yet to be successful in clinical trials (Supplementary Table 16). To refine the list of oncogenic pathways with significant differences in tumor efficacy and healthy tissue-of-origin tolerability, we compared dependency (TCGA_DEPMAP_) and tolerability (GTEX_DEPMAP_) scores across all genes and tissues (Fig. 7f). Pathway analysis of the strongest tumor dependencies with the least tissue-of-origin toxicity revealed enrichment of multiple oncogenic pathways and pathophysiological processes (Supplementary Table 17), including dysregulation of oxidative phosphorylation (*P* = 5.8 × 10^−11^) and mitochondrial translation (*P* = 2.9 × 10^−20^) pathways that were enriched in LUAD compared to healthy lung (Fig. 7g and Extended Data Fig. 5b). Combined, these observations suggest that predicted gene essentiality in the context of a driver mutation and correspondingly low essentiality within the healthy tissue of origin is likely to identify efficacious drug targets with acceptable tolerability.

### Tool for visualizing translational dependencies

To enable visualization of the data, we have provided an interactive web-based application (https://xushiabbvie.shinyapps.io/TDtool/) for exploring the data within TCGA_DEPMAP_, PDXE_DEPMAP_ and GTEX_DEPMAP_.

## Discussion

Cancer dependency maps have accelerated the discovery of tumor vulnerabilities, yet translating these findings to predict the therapeutic window of potential drug targets in patients remains challenging. Here, we used machine learning to build translational dependency maps in patient tumors and normal tissue biopsies that would enable tumor vulnerabilities to be studied in the context of a drug target’s efficacy, tolerability and outcome. The translational dependency maps were built using elastic-net models of transcriptomic features to predict gene essentiality. As the predictive models of essentiality did not include genomic features, the dependency scores could be independently tested for associations with genetic drivers in patient tumors. Moreover, these expression-only models of gene essentiality could be applied to healthy tissues that do not have appreciable levels of the somatic alterations that are observed in malignant tissues^28^. To illustrate how these data can be integrated to predict a target’s therapeutic window, we showed that *KRAS* and *BRAF* dependencies were elevated in patient tumors with GOF mutations (TCGA_DEPMAP_ and PDXE_DEPMAP_), which was far less pronounced in normal tissue biopsies lacking these driver mutations (GTEX_DEPMAP_). Combined, these new translational dependency maps offer a unique and clinically relevant aspect to gene essentiality that is not currently accessible in the traditional cell-based dependency maps. Finally, we made the dependency maps freely accessible in a user-friendly and interactive web-based application for exploring and visualizing the data.

**Extended Data Fig. 6:**
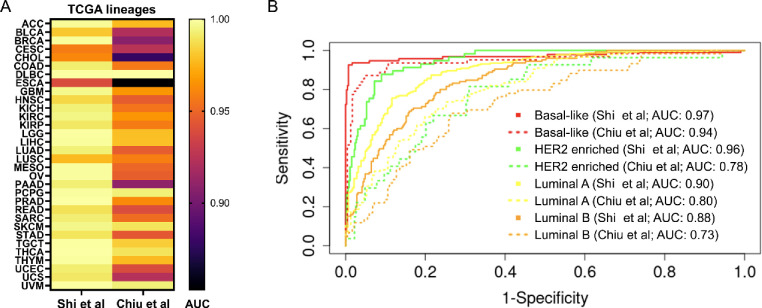
TCGA_DEPMAP_ outperforms DeepDEP. **a**) Precision-recall analysis of pan-cancer lineage predictions by the AUC values are significantly higher for TCGA_DEPMAP_ in predicting cancer lineages based on top 100 variable dependencies compared with DeepDEP. (**b**) The ROC curves for predicting the breast cancer subtypes based on the top 100 variable gene dependencies. The TCGA_DEPMAP_ significantly outperforms DeepDEP in predicting any of the breast cancer subtypes (TCGA_DEPMAP_ continuous line; DeepDEP dotted line). Source data

During the completion of this study, Chiu et al.^27^ took a complementary approach to building a translational dependency map (DeepDEP) using deep learning and the genomic, epigenomic and transcriptomic profiles of TCGA patients and DEPMAP cell lines. Here, we used elastic-net regularized regression models of expression data for predicting gene essentiality and tolerability, as these expression-based models performed comparably to multi-omics models and can be applied to malignant tissue (TCGA_DEPMAP_ and PDXE_DEPMAP_) and nonmalignant tissue (GTEX_DEPMAP_). The DeepDEP authors also highlighted that a simplified deep-learning model using expression only (Exp-DeepDEP) performed comparably well to DeepDEP^27^, suggesting that both approaches are dominated by expression data^27^. For lack of other ground truths, we compared the predicted tumor dependencies of TCGA_DEPMAP_ and DeepDEP by pan-cancer lineage and BRCA subtypes, as these were annotated by TCGA and DEPMAP. Compared to DeepDEP, the predicted dependencies by TCGA_DEPMAP_ were comparable in identifying cancer lineages and BRCA subtypes (Extended Data Fig. 6). Thus, the collective data demonstrated that the elastic-net models underlying TCGA_DEPMAP_, PDXE_DEPMAP_ and GTEX_DEPMAP_ performed well compared to DeepDEP. As additional studies become available, more in-depth benchmarking of approaches for translating dependencies is warranted, including the ability to detect genetic drivers, synthetic lethalities and other patient-relevant features.

A strength of translational dependency maps is the ability to recapitulate patient tumor context, therapeutic responses and many aspects of disease outcomes. Fitting with observations that the tissue of origin dominates the molecular landscape of cancer^53^, TCGA_DEPMAP_ and PDXE_DEPMAP_ revealed that tumor vulnerabilities were tightly correlated with disease lineage and subtype. Oncogenic dependencies were also predictive of response to molecularly targeted therapeutics in both TCGA_DEPMAP_ and PDXE_DEPMAP_, as would be expected based on the response rates for molecular therapeutics targeting oncogenic drivers in patients. In total, 85% of oncogenic dependencies had a GOF event associated with increased dependency in patient tumors and 28% could be associated with PFI, including some that predicted better or worse outcomes depending on the cancer lineage. These data fit with the observation that ~10% cancer-driver genes have evidence for both oncogenic and suppressive characteristics depending on tumor context. The selectivity of some oncogenic dependencies also differed between patients and cell models, including *FLT3, ATPV6V0E1* and *PTPN11*. Some of these discrepancies seemed to be attributed to cohort-specific distributions of the underlying drivers of SSDs (for example, *FLT3* and *ATPV6V0E1*), whereas others were likely attributable to different pathophysiological contexts, such as the 3D contexts of intact tumors versus the 2D contexts of cultured cells (for example, *PTPN11*). Taken together, these data highlight the complexities of interpreting gene essentiality in patient-relevant contexts, and future studies are warranted to further translate the underlying mechanisms of novel tumor dependencies that impact patient outcomes.

TCGA_DEPMAP_ detected multiple known synthetic lethalities (for example, *STAG1*/*STAG2*, *SMARCA2*/*SMARCA4* and *EP300*/*CREBBP*)^42,43,45,46^, as well as synthetic lethalities that are less well characterized (for example, *CNOT7*/*CNOT78* and *PAPSS1*/*PAPSS2*). As reported elsewhere^39,41,46^, synthetic lethal interactions varied widely when tested across different cancer cell models, suggesting that the currently available models are insufficient to account for all patient-relevant contexts. Nonetheless, both a commonly shared synthetic lethality (*CNOT7*/*CNOT78*) and a more selective synthetic lethality (*PAPSS1*/*PAPSS2*) were validated in vitro and in vivo. *CNOT7*/*CNOT78* are paralogous subunits of the CCR4–NOT complex that mediates messenger RNA stability^47^, fitting with the observation that loss of both subunits was broadly synthetic lethal. *PAPSS1*/*PAPSS2* are paralogous synthases of PAPS, which is required for sulfonation reactions^49^. We hypothesized that loss of *PAPSS2* is likely driven by its proximity to *PTEN* and is an example of collateral deletion in patient tumors^54^. This observation was confirmed by the synthetic lethal interaction of *PAPSS1* in UMUC3 cells that lacked *PAPPS2* and *PTEN*, which coincided with the inability of these cells to sulfonate proteins. Notably, the unique ability of TCGA_DEPMAP_ to detect and associate synthetic lethal mechanisms with patient outcomes revealed a worse OS of patients with an endogenous loss of *PAPSS2* and a predicted synthetic lethality with *PAPSS1* dependency. Thus, these data collectively highlight the benefits of translational dependency maps that closely match the pathophysiological contexts of intact patient tumors and the diversity of patient genomic datasets to identify clinically relevant mechanisms^1,55^.

A unique aspect of this study was the ability to systematically compare gene essentiality associated with somatic mutations in TCGA_DEPMAP_ with the healthy tissue-of-origin tolerability profiles in GTEX_DEPMAP_. Systematically expanding this analysis across all gene essentiality models in TCGA_DEPMAP_ and GTEX_DEPMAP_ revealed wide variability in the predicted tolerability windows, implicating the existence of other dependencies with strong genetic drivers that are likely to be more tolerable as therapeutic targets; however, when interpreting these data, we also recommend exercising caution, as the tolerability windows predicted by comparing tissue-of-origin gene essentiality between TCGA_DEPMAP_ and GTEX_DEPMAP_ likely does not yet fully capture the other dose-limiting toxicities that pose challenges to clinical drug development^56^. As such, future efforts to model gene essentiality in healthy tissues should expand to incorporate systems approaches to integrating tolerability signals across multi-organ physiological pathways and systems.

The translational dependency maps presented in this study provide insights into gene essentiality and tolerability in the clinical context of patient tumors and healthy tissues. The ability of these maps to accurately translate dependencies to patients is reliant on the ability to build predictive models from cell-based mapping, which is still at the early stages and is expected to require 20× more data to fully predict gene essentiality^7^. Further, the observations that cell-based dependencies vary between 2D and 3D settings^57^ and are impacted by crosstalk with the tumor microenvironment^58^, suggests that gene essentiality is contextual and requires models with greater relevance to intact tumors, such as organoids. Likewise, it is equally plausible that accurately interpreting translational dependencies will require a deeper understanding of clonal heterogeneity with patient tumors that is lacking from homogenous cancer cell lines. To reach the full potential of translational dependency mapping, the catalog of patient genomic datasets will also likely require expansion to capture various stages of disease progression, including tumorigenesis^2^, metastasis^3,59^ and therapeutic resistance^3,4,59^. Furthermore, as precision cancer clinical trials continue to expand (for example, MSK-IMPACT)^4^, it will be increasingly possible to refine translational dependency maps by testing outcomes of molecular therapeutics with predicted target essentiality. The utility of translational ‘tolerability’ maps in healthy tissues (for example, GTEX_DEPMAP_) remains to be fully explored and will likely benefit from further refinements to better capture aspects of dose-limiting toxicities that impact drug development. To this end, we postulate that modeling gene tolerability could be best assessed in normal cell types by pairing CRISPR perturbations with single-cell RNA sequencing^60,61^ to broadly capture the alterations of pathways required for healthy tissue homeostasis. Ultimately, we postulate that predictive modeling of dependency and tolerability in patients will increase the success of drug discovery by preemptively prioritizing targets with the best therapeutic index (high dependency and tolerability).

## Methods

### Predictive modeling of gene essentiality using DEPMAP data

**Extended Data Fig. 7:**
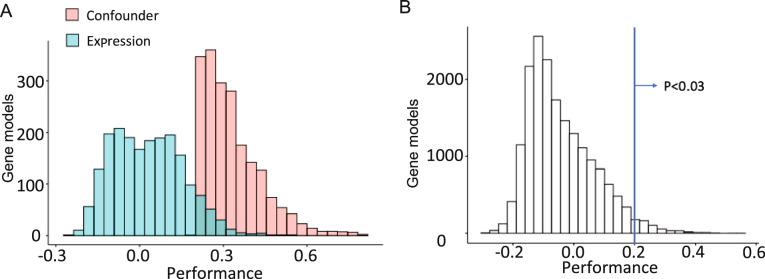
Comparison of cross-validated models with models generated using the DepMap confounders dataset as a null distribution, including sex, cas9 activity, age, lineage, primary or metastasis, growth pattern, library, screen quality and cancer type. (**a**) Distribution of model performance across expression-only and confounder models. (**b**) The expression-only gene essentiality models significantly outperformed the models built on confounders, with the 0.2 cross-validation threshold corresponding to p < 0.03 in the confounder distribution (~7000 models). Source data

Two sets of elastic-net regression models were generated to predict gene essentiality from the DEPMAP (*n* = 897 cell lines) with RNA alone (expression only) or combined with mutation and copy number profiles (multi-omics). Gene effect scores were estimated by CERES^24^, which measures the dependency probability of each gene relative to the distribution of effect sizes for common essential and nonessential genes within each cell line^25^. Because many genes do not impact cell viability (CERES < −0.5), elastic-net models were attempted only for genes with at least five dependent and nondependent cell lines, which included 7,260 out of 18,119 genes (40%) with effects scores in the DEPMAP (1Q21 release). Genome-wide datasets (19,005 genes) for RNA-seq, mutations and copy number variants (log_2_ relative to ploidy + 1) for the 897 cell lines were downloaded directly from the DEPMAP (1Q21; https://depmap.org/portal/). The ‘glmnet’ package (v.4.1.3)^23^ was used to build elastic-net regularized regression models with balanced weights for L1 and L2 norm regularization. The α values were kept constant at 0.5 for all models. Models were tenfold cross-validated using ‘lambda.min’ from cv.glmnet from the glmnet R package (100 lambdas tested per model by default) to select the lambda showing the minimum error balanced with the prediction performance and the number of features selected, as described previously^61^. The performance of the optimal model was then assessed by Pearson’s correlation coefficient (*R*), with a ‘pass’ threshold of *R* > 0.2 and FDR < 0.001 to correct for multiple hypothesis testing. The cross-validated models were also compared to models generated using the DepMap confounders dataset as a null distribution, including sex, cas9 activity, age, lineage, primary or metastasis, growth pattern, library, screen quality and cancer type. As shown in Extended Data Fig. 7, the expression-only gene essentiality models significantly outperformed the models built on confounders, with the 0.2 cross-validation threshold corresponding to *P* < 0.03 in the confounder distribution (~7,000 models). Cross-validation confirmed 1,966 expression-only models and 2,045 multi-omics models, of which the majority of cross-validated models overlapped (*n* = 1,797) between the two datasets (Supplementary Table 3).

### Model transposition following transcriptional alignment of DEPMAP to TCGA, PDXE and GTEX datasets to build TCGA_DEPMAP_, PDXE_DEPMAP_ and GTEX_DEPMAP_

The translational dependency maps TCGA_DEPMAP_, PDXE_DEPMAP_ and GTEX_DEPMAP_ were built using expression-only models of gene essentiality, based on relatively marginal performance gains in the multi-omics models of gene essentiality, as reported elsewhere^26,27^. To enable transposition of the cross-validated expression-only models (*n* = 1,966) from the DEPMAP to TCGA (*n* = 9,596 tumors), PDXE (*n* = 191 tumors) and GTEX (*n* = 17,382 tissues across 54 tissues and 948 donors), the genome-wide gene expression datasets were downloaded for TCGA (https://xenabrowser.net/datapages/), PDXE^50^ and GTEX (https://gtexportal.org/home/datasets). For TCGA data, if multiple samples were collected from the same patient, only the primary tumor biopsy was included in TCGA_DEPMAP_. For GTEX, the potential biases introduced by sampling multiple organ tissues from each individual was assessed by Uniform Manifold Approximation and Projection (UMAP) analysis of the gene expression profiles across GTEX samples, which revealed that GTEX samples are clustered by tissue types rather than by individuals. Likewise, no evidence of clustering was observed based on other patient-specific clinical variables (for example, cause of death and age), suggesting that the tissue-specific effects are the predominant drivers of gene expression in healthy tissues.

Unsupervised cluster analyses by UMAP dimension reduction were used to evaluate the similarities in expression profiles of the DEPMAP cell lines compared to the tissue biopsies from TCGA, PDXE and GTEX. As reportedly previously^56^, DEPMAP and TCGA expression profiles do not cluster well by UMAP alignment due to contaminating transcriptional profiles of stromal and immune cells, which would impact expression-based predictive modeling of gene essentiality. Likewise, UMAP clustering of expression profiles from the DEPMAP cell line data compared to PDXE and GTEX samples revealed that transcriptional alignment of these data was equally problematic. To overcome this issue, expression data from DEPMAP and TCGA were quantile normalized and transformed by cPCA, which is a generalization of the PCA that detects correlated variance components that differ between two datasets. When comparing the transcriptional profiles of the DepMap cell lines and TCGA patient tumors, the top contrastive principal components (cPC1–4) derived from the stromal contamination in TCGA, which were then removed followed by multiple-batch correction to normalize the expression data by matching the corresponding clusters in TCGA and DEPMAP. To assess transcriptional alignment on model transposition, all pre- and post-aligned TCGA_DEPMAP_ gene essentiality models were compared to tumor purity, which revealed a strong correlation between gene essentiality and tumor purity that was removed by transcriptional alignment. An identical approach was utilized for aligning PDXE expression data, with the slight modification that only cPC1–3 required removal, as PDX models grown in immunocompromised mice lack the adaptive immune system and typically have lower stromal contamination. For aligning DEPMAP and GTEX data, a slightly different approach was used to combine quantile normalization and ComBat^62^ to remove potential batch effects without using cPCA, as GTEX data only includes nonmalignant tissue. Finally, the observed (DepMap) and predicted (TCGA_DEPMAP_, PDXE_DEPMAP_ and GTEX_DEPMAP_) gene essentiality scores were aligned by linear regression, whereby the slopes of each model were fitted using a constant to make the absolute value comparable to the measured essentiality values. Notably, because this approach used a scaling factor, the pattern of gene essentiality scores was not affected. All data are available on figshare^63^.

### Characterization of TCGA_DEPMAP_

The distribution of the cross-validated expression-only models of gene essentiality (*n* = 1,966) across lineages was assessed by unsupervised cluster analysis (Ward.D2 method) and visualized using the ComplexHeatmap R package (v.2.6.2). A similar approach was used for unsupervised cluster analysis and heatmap visualization for molecular subtyping of the BRCA cohort of TCGA_DEPMAP_ using the DEP100 across BRCA cohort only. For lack of other ground truths, the performance of TCGA_DEPMAP_ to classify molecular subtypes of BRCA was benchmarked using a linear discriminant analysis with leave-one-out cross-validation performed using the MASS package (v.7.3.51.4) for R and the CV = TRUE option in the function. Predictions for each cancer type and subtype was evaluated separately and the AUC values were determined using the function ‘roc’ from the pROC (v.1.18.0) package for R and compared to the molecular typing and subtyping reported by the TCGA (https://www.cbioportal.org/)^64^. In addition to BRCA molecular subtypes, a distinct subset of the 100 most variable dependencies from the pan-cancer TCGA_DEPMAP_ dataset was used to benchmark TCGA_DEPMAP_ more broadly, using an identical linear discriminant analysis with leave-one-out cross-validation, as described above. Finally, both analyses were repeated with the DeepDEP gene essentiality values reported by Chiu et al.^27^ and the receiver operating characteristic (ROC) AUC values were compared between TCGA_DEPMAP_ and DeepDEP predictions of cancer lineages and BRCA cancer subtypes.

Associations of dependencies with genomic features (somatic mutations and copy number variants) in TCGA_DEPMAP_ were assessed using a Wilcoxon rank-sum differential test as implemented using stat_compare_means function of ggpubr R package (v.0.4.0). The ability of expression features to predict essentiality and mutational status of same gene by elastic-net modeling was compared using the glmnet R package (v.4.1) with the same parameters for both model sets. The elastic-net models were allowed to select the most informative predictive features for mutation and essentiality for each gene, as the best predictors for essentiality may not be the best features to predict mutation. For AUC evaluation, we used −0.5 as the cutoff for gene essentiality scores to determine sensitive and resistant cells for gene models. The AUC values are calculated using pROC R package (v.1.16.2). To characterize SSDs, a normality likelihood ratio test (NormLRT)^29^ was performed with slight modifications to rescale the larger NormLRT values observed in TCGA_DEPMAP_ due to a tenfold larger cohort size (*n* = 9,596) compared to DEPMAP (*n* = 897). A bootstrapping of the DEPMAP gene effect scores was performed to estimate how the NormLRT scores change when scaling up from the DEPMAP cohort size (*n* = 897 cell models) to the cohort size of TCGA (9,596). A linear fitting was performed to estimate the slope between DEPMAP and bootstrapped equivalent, which was as a scaling factor (0.07) to rescale TCGA NormLRT scores. Notably, outliers were identified based on the ranking NormLRT scores within each cohort, which therefore was not affected by the rescaling TCGA NormLRT scores. For TCGA patients with BRCA (*n* = 765), we divided the patients into *PTPN11* dependent and nondependent groups. The *PTPN11*-dependent patients (77 patients) were selected as the top 10% patients with BRCA with the lowest *PTPN11* essentiality scores. Among all the variants, we applied Fisher’s exact test for mutations with more than 5% frequency (12 mutations), deletions with more than 10% frequency (4,891 deletions) and amplifications with more than 10% frequency (4,831 amplifications). The test was performed using the fisher.test function in the stats (v.4.0.3) R package with options ‘alternative = greater’ to calculate *P* values for enrichment of variants for *PTPN11* dependent and nondependent groups. The gene models (890 models) used for mutation predictions are selected from 1,966 cross-validated expression-only essentiality models with a mutation frequency >2%.

### Associating clinical outcomes with tumor dependencies in TCGA_DEPMAP_

Owing to the limited accessibility of therapeutic response data in TCGA^33^, the association of *HER2* essentiality with response to trastuzumab (anti-HER2 antibody) was tested in a recent trastuzumab clinical trial of 50 *HER2*^+^ patients with BRCA with pre- and post-treatment biopsies that were analyzed by microarray^34^. The microarray expression data were downloaded from NCBI GEO (accession code GSE76360) and patient responses were defined by the study authors^34^. Differences in predicted *HER2* essentiality in patients with different clinical responses were tested using ggpubr R package (v.0.4.0), followed by a Wilcoxon rank-sum test using the stat_compare_means function in the package. Correlation of *HER2* essentiality and HER2 expression after treatment was tested by a Pearson’s correlation, as calculated by the stat_cor function ggpubr R package (v.0.4.0). For predicting essentiality response to sorafenib, although it is a multi-kinase inhibitor (BRAF, CRAF, VEGFR2, VEGFR3, PDGFRB, FLT3 and cKIT), its role in treating hepatocellular carcinoma (HCC) is widely attributed to inhibiting oncogenic RAF signaling. This combined with the observation that *BRAF* essentiality model performance (*R* = 0.71) was far better than the other target models (*R* = 0.2 to 0.45), led us choose the *BRAF* essentiality model to predict sorafenib response in the HCC cohort.

Additionally, the correlation of TCGA_DEPMAP_ dependencies with the PFI of TCGA patients was performed, excluding the acute myeloid leukemia (AML), diffuse large B-cell lymphoma (DLBC), kidney chromophobe (KICH) and pheochromocytoma and paraganglioma (PCPG) cohorts based on the recommendations of Liu et al.^33^. The PFI data were directly downloaded from Liu et al.^33^ and the maximally selected rank statistics from the ‘maxstat’ R package was used to determine the optimal cutoff point for dichotomization (high versus low) of dependency scores (*n* = 1,966 cross-validated models). The prognostic value of the resulting dichotomized dependency scores was evaluated using the log-rank test with FDR correction (Benjamini–Hochberg adjusted) to account for multiple hypothesis testing. The data were visualized by Kaplan–Meier curves and are interpreted as HR > 1 indicating a worse expected outcome in patients with a higher dependency score at an FDR < 0.2.

### Predicting synthetic lethality relationships in TCGA_DEPMAP_

Multiple approaches were integrated to predict and prioritize synthetic lethality relationships with LOF events (defined as a predicted copy number loss or damaging mutation) in TCGA_DEPMAP_. Lasso regression was used to identify gene essentialities (*n* = 7,260 expression-only models) with increased dependencies associated with 25,026 LOF events in TCGA, as annotated by Bailey et al.^65^. For each model, the lambda value was selected as the lowest error by fivefold cross-validation and the resulting models with coefficients >0.3 were further evaluated by a *t*-test. The lasso regression analysis identified 633,232 predicted synthetic lethal candidates (FDR < 0.01), which were too numerous to experimentally validate and required further prioritization. First, UNCOVER^66^ was used to prioritize synthetic lethal candidates predicted by TCGA_DEPMAP_ that correlated with endogenous mutual exclusivity of LOF mutations (3–70% prevalence) in TCGA, with the hypothesis that these candidates would have greater translational relevance. UNCOVER was ran in greedy mode (UNCOVER_greedyv2.py) to identify negative association with a mutated gene sets of maximum ten genes. To evaluate the confidence of association, we set the number of permutations as 100 to compute *P* values and applied a threshold of *P* < 0.01. Of the 633,232 predicted synthetic lethal candidates predicted by TCGA_DEPMAP_, 28,609 pairs also had evidence of mutually exclusive mutation rates in TCGA. The candidate list was then refined further by prioritizing paralogs using the biomaRt paralog database (v.2.28.0) R package. We additionally included pairs characterized by phylogenetic distance with threshold less than 1.5, as described previously^67,68^. The candidate list received a final filtering based on overall patient prevalence of LOF events, protein–protein interactions with TSGs^69,70^, previous experimental evidence of gene–gene interactions^6,16,17,39,40^ and manual curation to include essential and nonessential controls. The final list of gene pairs that were prioritized for experimental validation included 601 synthetic lethality candidates from the original lasso regression of TCGA_DEPMAP_ and an additional 264 pairs that were retained as library controls. The list of all synthetic lethal pairs that were predicted by TCGA_DEPMAP_, as well as annotations of mutual exclusivity and phylogenetic distance, is provided as an R object in the figshare repository (https://figshare.com/s/a76d338a425273b42c8b)^71^.

### Multiplexed screening synthetic lethalities using AsCas12a (AsCpf1) and enAsCas12a (enAsCpf1)

Guides were designed using the TTTV PAM for AsCas12a and synthesized into four-guide arrays with direct repeats (DR)−0, −1, −2 and −3 preceding each guide, followed by cloning into a guide-only lentiviral vector (pRDA_052), as described previously^45,46^. A DKO construct was designed with two guides × two genes (*n* = 4 guides total per construct) for each pair of synthetic lethal candidates. Single KO constructs were also designed two guides × one gene + two nontargeting (NT) guides (*n* = 4 guides total per construct) for each pair of synthetic lethal candidates. For some pairs, multiple single KOs were used to assess overall library variance and were collapsed to the median values for downstream gene interaction analysis. A total of 500 constructs with four NT guides were also included in the library as negative controls. An initial set of pilot screens were performed in triplicate using A549 (ATCC), NCI-H1299 (ATCC), MDA-MB-231 (ATCC), PC3M (MD Anderson) and DETROIT562 (ATCC) that stably express AsCas12a, as described previously^46^. An enhanced AsCas12a (enAsCas12a) enzyme was recently reported that is compatible with CRISPR/AsCas12a libraries^44^, enabling an independent replication of the initial pilot screens and expansion to a total 14 total cancer cell models. The subsequent screens using enAsCas12a were performed in triplicate using A549 (ATCC), NCI-H1299 (ATCC), MDA-MB-231 (ATCC), NCI-H1703 (ATCC), PC3M (MD Anderson), DETROIT562 (ATCC), HT29 (ATCC), HCT116 (ATCC), PANC1 (ATCC), MIAPACA2 (ATCC), SNU1 (ATCC), HSC2 (JCRB), HSC3 (JCRB) and FADU (ATCC). For all screens, cells were infected at a multiplicity of infection of 0.3 and cultured for 14 days while continuously maintaining 500× coverage, followed by DNA extraction and PCR-barcoding using the p5 Agon and p7 Kermit primers^46^. The PCR-barcoded libraries were single-end sequenced using an Illumina HiSeq4000 (300× cycle), followed by demultiplexing of sequencing reads (bcl2fastq, Illumina) and quantification of guide array abundance across all samples was performed with a custom Perl script. Sequences between the flanking sequences or by location were extracted and compared to a database of sgRNA for each library. Only perfectly matched sgRNA sequences were kept and used in the generation of count matrix. Normalization between all samples was conducted using the ‘TMM’ method^72^ implemented in the edgeR R Bioconductor package. The log_2_ fold changes (L2FCs) of guide array abundance were calculated by comparing day 14 libraries with the plasmid library using limma-voom^73^. GIs were calculated by comparing the expected and observed L2FC of double and single KO constructs, as described previously^39,45^. In brief, the expected L2FC for DKO constructs is calculated as a sum (LF2C) of the individual knockout (sgRNA + NT). Synthetic lethal and buffering interactions are defined for DKO in which the observed double knockout L2FC is significantly greater or less than that of the expected L2FC, respectively. No statistical methods were used to predetermine sample sizes but our sample sizes are similar to those reported in previous publications that have used multiplexed CRISPR to screen synthetic lethal interactions^39,45^.

### Experimental validation of *PAPSS1*/*2* and *CNOT7*/*8* synthetic lethality

**Extended Data Fig. 8:**
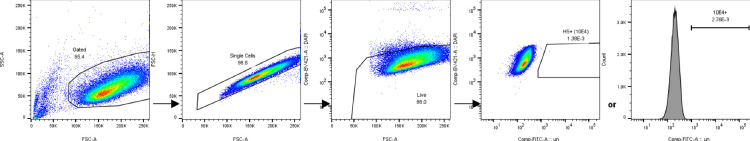
Gating strategy for Flow Cytometry plots. Cells were first gated by FSC-A/SSC-A (~95%), and single cells by FSC-A/FSC-H (~98%). DAPI staining was used to gate viable cells (~98%). Unstained cells and/or Heparinase III treated cells were used for establishing the positive 10E4-FITC gate. Source data

CRISPR/Cas12 KOs of *PAPSS1*, *PAPSS2*, *CNOT7* and *CNOT8* were performed with Cas12 Ultra (Integrated DNA Technologies, 10007804) according to the manufacturer’s instructions by Neon electroporation of RNPs (Invitrogen). Guides were designed using the Broad Institute CRISPick algorithm and the two best-performing guides for each gene were used in combination (Supplementary Data). Protein expression was quantified by Simple Western (ProteinSimple, BioTechne) using the following antibodies; PAPSS1 clone 1F4 (Abnova, H00009061-M05) at 1:100 dilution, PAPSS2 (Cell Signaling Technology, 70638) at 1:50 dilution, PTEN (Cell Signaling Technology, 9552) at 1:100 dilution, CNOT7 (Santa Cruz, sc-101009) at 1:10 dilution, CNOT8 (LSBio, LS-C99242-400) at 1:1,000 dilution with β-actin clone 8H10D10 (Cell Signaling Technology, 3700), 1:1000 GAPDH clone 14C10 (Cell Signaling Technology, 2118) or 1:1,000 α-tubulin (Cell Signaling Technology, 2144) as loading controls. Flow cytometry analysis of sulfonated HSPGs was performed with the 10E4 antibody conjugated to FITC and used at 1:200 dilution (US Biological Life Sciences, H1890-10) (Extended Data Fig. 8). Bacteroides heparinase III was obtained from New England Biolabs (P0737L) and used as per manufacturer’s protocol by treating cells for 1 h in reaction buffer at 30 °C before FACS analysis. Spheroid cultures were performed on ultra-low attachment 96-well plates (Corning, 7007), growth was tracked on Incucyte S3 (Sartorius) and CellTiterGlo (CTG) readouts were performed for viability measurements (Promega, G9681). For rescue experiments, HS was used at 10–50 μg ml^−1^ (Sigma, H7640). For CNOT7-null single-clone generation, HT29 (ATCC) cells were transduced with pFUN_104 Cas9 plasmid (Broad Institute), CNOT7-KO was performed with CRISPR/Cas12 RNP electroporation, CNOT7-KO single clones were isolated and expanded and clones sc2 and sc7 were transduced with the Cellecta pRSGTEP-U6Tet-sg-EF1-TetRep-2A-Puro vector containing the CNOT8-targeting sgRNA (sgCNOT8; 5′-CCAGGTTATCTGTGAAGTGT-3′ (CVCRC-PX, 98847-3P) or NT control (sgNT; 5′-GGCAGTCGTTCGGTTGATAT-3′ SGCTL-NT-pRSGTEP). Cells were then cultured in medium containing Tet System Approved FBS (TakaraBio, 631101) and dox was used at 1 μg ml^−1^ for in vitro experiments. For in vivo experiments, 1 × 10^6^ UMUC3 (ATCC) or HT29 cells were reconstituted in Hanks balanced salt solution, mixed 1:1 with Matrigel (Corning, 356235) and 200 μl inoculated in the right flank (*n* = 5 mice per condition). Female CB17/SCID and SCID/beige at 6–8 weeks of age were obtained from Charles River. In vivo experiments were conducted in compliance with AbbVie’s Institutional Animal Care and Use Committee and the National Institutes of Health guidelines in the Health Guide for Care and Use of Laboratory Animals. Tumor measurements of length (*L*) and width (*W*) were obtained using calipers and volume (*V*) calculated using the formula *V* = (*L* × *W*^2^)/2. A maximum of 2,000 mm^3^ tumor volume was allowed as per institutional guidelines. *PAPSS1*/*PAPSS2* tumors were extracted at day 22, mechanically dissociated with scalpels and single-cell suspensions were made using Liberase and DNase I (Millipore Sigma, 05401127001 and 11284932001, respectively) incubated at 37 °C for 1 h and mouse cells were magnetically depleted on LS columns using mouse cell depletion cocktail (Miltenyi, 130-104-694 and 130-042-401). No statistical methods were used to predetermine sample sizes but our sample sizes are similar to those reported in previous publications that have tested tumor vulnerabilities and synthetic lethalities^16,42,43,74,75^.

### Characterization of PDXE_DEPMAP_

The distribution of the cross-validated expression-only models of gene essentiality (*n* = 1,966) across lineages was assessed by unsupervised cluster analysis (Ward.D2 method) and visualized using the ComplexHeatmap R package (v.2.6.2). Associations of dependencies with genomic features were assessed using a Wilcoxon rank-sum differential test as implemented using stat_compare_means function of the ggpubr R package (v.0.4.0). To test the ability of gene essentiality to predict the response to corresponding targeted therapies, the change in PDX burden from baseline to experimental end point was correlated with target gene essentiality in PDXE_DEPMAP_ using a Pearson’s correlation test and FDR correction of *P* values for multiple hypothesis testing. ROC AUC analysis was performed using the pROC R package (v.1.18.0) to assess the accuracy of drug responses predicted by the target gene essentiality scores. Only drugs with at least 20 treated PDX models were evaluated and the metrics are reported in Supplementary Table 13.

### Characterization of GTEX_DEPMAP_

**Extended Data Fig. 9:**
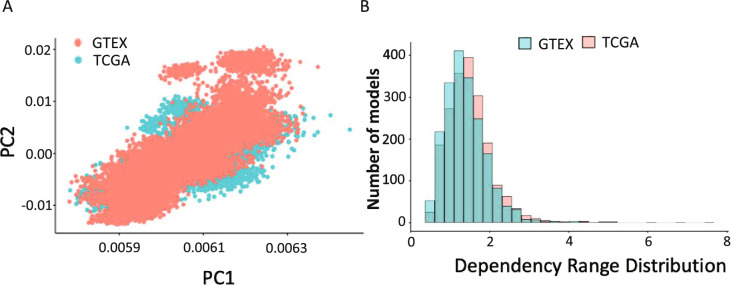
The GTEX and TCGA expression profiles were aligned and normalized independently to the same DepMap expression profile and the same models (genes and coefficients) were used for both datasets. (**a**) Overall range of effect sizes for both datasets was investigated using a PCA, which demonstrates that the dependency distributions show that the predicted dependency scale is very similar for the two datasets. (**b**) The distribution of gene essentiality scores is similar between TCGA_DEPMAP_ and TCGA_DEPMAP_. Source data

The distribution of the cross-validated expression-only models of gene essentiality (*n* = 1,966) across healthy tissues was assessed by unsupervised cluster analysis (Ward.D2 method) and visualized using the ComplexHeatmap R package (v.2.6.2). Differences in gene essentiality in healthy and malignant tissues, as well as malignant tissues with genomic features, were assessed using a Wilcoxon rank-sum differential test as implemented using stat_compare_means function of ggpubr R package (v.0.4.0). Notably, the distributions of dependencies between TCGA_DEPMAP_ and GTEX_DEPMAP_ by PCA revealed that that the predicted dependency scales are similar between the two datasets (Extended Data Fig. 9) and thus any differences in gene essentiality are due to underlying biological mechanisms that differ between healthy and malignant tissues. To evaluate the sensitivity and specificity of GTEX_DEPMAP_ to genes associated with tissue-specific toxicities, we profiled GTEX_DEPMAP_ genes associated with both blood disorders and drug-induced liver toxicity using the Cortellis OFF-X database (https://targetsafety.info/). The OFF-X database is a drug and target safety intelligence database that predicts potential associations based on both preclinical and clinical safety data alerts from peer-reviewed journals, company communications, clinical trials and regulatory agency communications. These blood and liver toxicity associations were further evaluated to identify overlapping or unique genes for each toxicity and annotated with the frequency of associated safety alerts. In total, the Cortellis OFF-X database identified drug targets associated with potential toxicities in blood (*n* = 82), liver (*n* = 85) or blood and liver (*n* = 74), which were then compared across healthy tissue lineages in GTEX_DEPMAP_. To compare gene essentiality between malignant and healthy tissues, TCGA_DEPMAP_ and GTEX_DEPMAP_ samples were matched based on the tissue of origin and a Student’s *t*-test was applied to differential analysis between the dependency profiles of tumor and healthy tissue of the same lineages. The *t*-statistic was used to characterize the dependency difference between the tumor and corresponding healthy tissue with a negative *t*-statistic value corresponding to a higher dependency in tumor as compared to the healthy tissue. Gene set enrichment analysis was performed across all paired malignant and healthy tissues of origin. The list of genes for the lung network was generated using the top 100 genes showing the largest differentiation in gene essentiality between cancer compared to healthy tissue in lung based on the negative *t*-statistic values. Network connectivity and gene ontology enrichment were calculated using STRING (https://string-db.org/), as described previously^76^.

### Statistics and reproducibility

All data used for the machine learning and translation of gene essentiality are from publicly available consortia with detailed methodologies for data collection, blinding, randomization and protection. Because the essentiality profiles have a long tail distribution, we have used the nonparametric Wilcoxon test, which does not require a particular probability distribution of the dependent variable in the analysis. Therefore, no tests were required for the normality assumption. No statistical method was used to predetermine sample size. No data were excluded from the analyses. The experiments were not randomized. The investigators were not blinded to allocation during experiments and outcome assessment.

### Reporting summary

Further information on research design is available in the Nature Portfolio Reporting Summary linked to this article.

### Supplementary information

Reporting Summary

Supplementary Data 1 Sequence for the CRISPR guide and SL library.

Supplementary Table 1 Supplementary Table 1: expression-only elastic-net model coefficients of gene essentiality predicted from the DEPMAP. The rows denote features used in modeling and columns are corresponding gene models.

Supplementary Table 2 Supplementary Table 2: multi-omics elastic-net model coefficients of gene essentiality predicted from the DEPMAP. The rows denote features used in modeling and columns are corresponding gene models.

Supplementary Tables 3–17 Supplementary Table 3: list of cross-validated genes from expression-only and multi-omics models. Supplementary Table 4: pathway enrichment analysis of dependencies most changed by the transcriptional alignment, as defined by gene essentiality scores with the least correlation (*r* < 0.5) before and after alignment of DEPMAP and TCGA transcriptomes. Supplementary Table 5: gene essentiality scores across TCGA_DEPMAP_. The row labels denote the gene model and the column labels correspond to TCGA patient sample ID (https://figshare.com/projects/TCGADEPMAP_Mapping_Translational_Dependencies_and_Synthetic_Lethalities_within_The_Cancer_Genome_Atlas/130193). Supplementary Table 6: association of oncogene essentiality with GOF events. Supplementary Table 7: SSDs across DEPMAP and TCGA_DEPMAP_, as assessed by the NormLRT. Supplementary Table 8: predicting therapeutic response in clinical datasets based on the target’s dependency profiles. Supplementary Table 9: Cox log-rank analysis of gene essentiality associated with PFI in TCGA_DEPMAP_. The row labels denote the gene models and the column heads correspond to the HRs and *P* values, respectively. Supplementary Table 10: annotation of synthetic lethality candidates that were experimentally tested by multiplexed CRISPR/Cas12 screening. The row labels denote gene pairs and column labels correspond to whether a pair was predicted by TCGA_DEPMAP_ and whether the pair was annotated as paralogs or TSGs. Supplementary Table 11: GI scores from CRISPR/Cas12 screens using AsCas12a and enAsCas12a across 14 cell models. The row labels denote the gene pairs and the column labels correspond the to the enzyme used (AsCas12a or enAsCas12a), the expected (exp.) effect (summed *z*-score of the single KO), the observed (obs.) effect of DKO (z-score transformed), the difference (Diff.) of expected and observed effects and the *z*-score transformation of the difference (Diff_Z). Supplementary Table 12: gene essentiality scores across PDXEDEPMAP. The row labels denote the gene model and the column labels correspond to the PDX sample ID (https://figshare.com/projects/TCGADEPMAP_Mapping_Translational_Dependencies_and_Synthetic_Lethalities_within_The_Cancer_Genome_Atlas/130193). Supplementary Table 13: predicting drug response based on gene essentiality in PDX models. Supplementary Table 14: gene essentiality scores across GTEX_DEPMAP_. The row labels denote the gene model and the column labels correspond to the GTEX sample ID (https://figshare.com/projects/TCGADEPMAP_Mapping_Translational_Dependencies_and_Synthetic_Lethalities_within_The_Cancer_Genome_Atlas/130193). Supplementary Table 15: list of genes associated with both blood disorders and drug-induced liver toxicity that were curated from the Cortellis OFF-X database. The OFF-X database is a drug and target safety intelligence database that predicts potential associations based off both preclinical and clinical safety data alerts from peer-reviewed journals, company communications, clinical trial and regulatory agency communications. The rows denote the genes associated with potential toxicities in blood (*n* = 82), liver (*n* = 85) or blood and liver (*n* = 74). The column labels correspond to the different evidence classes of safety alerts. Supplementary Table 16: differences between predicted essentiality between malignant tissue and the healthy tissue of origin. The rows denote the gene model and the column labels correspond to the malignant (TCGA_DEPMAP_) and healthy (GTEX_DEPMAP_) tissue types that were matched based on the tissue of origin. A Student’s *t*-test was applied to differential analysis between the dependency profiles of tumor and healthy tissue of the same lineages. The *t*-statistic was used to characterize the dependency difference between the tumor and corresponding healthy tissue with a negative *t*-statistic value corresponding to a higher dependency in tumor as compared to the healthy tissue. Supplementary Table 17: pathway analysis of the strongest tumor dependencies with the least normal tissue-of-origin toxicity. The rows denote the pathway and the column labels correspond to the enrichment *P* value, FDR-corrected *P* value (*P*adj), normalized enrichment score, number of genes included in the gene set (size), the gene models comprising the leading edge (leadingEdge) and the paired malignant and healthy tissues (type).

### Source data

Source Data Fig. 1 Source data for Fig. 1.

Source Data Fig. 2 Source data for Fig. 2.

Source Data Fig. 3 Source data for Fig. 3.

Source Data Fig. 4 Source data for Fig. 4.

Source Data Fig. 5 Source data for Fig. 5.

Source Data Fig. 6 Source data for Fig. 6.

Source Data Fig. 7 Source data for Fig. 7.

Source Data Fig. 4 Uncropped image for Fig. 4h.

Source Data Extended Data Fig. 1 Source data for Extended Data Fig. 1.

Source Data Extended Data Fig. 2 Source data for Extended Data Fig. 2.

Source Data Extended Data Fig. 3 Source data for Extended Data Fig. 3.

Source Data Extended Data Fig. 4 Source data for Extended Data Fig. 4.

Source Data Extended Data Fig. 5 Source data for Extended Data Fig. 5.

Source Data Extended Data Fig. 6 Source data for Extended Data Fig. 6.

Source Data Extended Data Fig. 7 Source data for Extended Data Fig. 7.

Source Data Extended Data Fig. 8 Source data for Extended Data Fig. 8.

Source Data Extended Data Fig. 9 Source data for Extended Data Fig. 9.

Source Data Extended Data Fig. 10 Uncropped images for Extended Data Fig. 10.

## Data Availability

All data are available in the supplementary information and the figshare repository at https://figshare.com/projects/TCGADEPMAP_Mapping_Translational_Dependencies_and_Synthetic_Lethalities_within_The_Cancer_Genome_Atlas/130193 (ref. ^63^). Source data are provided with this paper.

## Code availability

The R scripts used to download, format and analyze the data and produce the interactive R/Shiny app are available on GitHub (https://github.com/xushiabbvie/TDtool).
