# Supplementary material for: Building a translational cancer dependency map for The Cancer Genome Atlas
Source: Nat Cancer. 2024 Jul 15;5(8):1176–94. doi: 10.1038/s43018-024-00789-y (PMC11358024; doi:10.1038/s43018-024-00789-y)
Supplement: Supplementary file 1 — Reporting Summary [file 43018_2024_789_MOESM1_ESM.pdf]

nature portfolio

Corresponding author(s): NATCANCER-A08706B

Last updated by author(s): Jan 8, 2024

Reporting Summary

Nature Portfolio wishes to improve the reproducibility of the work that we publish. This form provides structure for consistency and transparency in reporting. For further information on Nature Portfolio policies, see our Editorial Policies and the Editorial Policy Checklist.

Statistics

For all statistical analyses, confirm that the following items are present in the figure legend, table legend, main text, or Methods section.

n/a	Confirmed	
☐	☒	The exact sample size (*n*) for each experimental group/condition, given as a discrete number and unit of measurement
☐	☒	A statement on whether measurements were taken from distinct samples or whether the same sample was measured repeatedly
☐	☒	The statistical test(s) used AND whether they are one- or two-sided<br>*Only common tests should be described solely by name; describe more complex techniques in the Methods section.*
☐	☒	A description of all covariates tested
☐	☒	A description of any assumptions or corrections, such as tests of normality and adjustment for multiple comparisons
☐	☒	A full description of the statistical parameters including central tendency (e.g. means) or other basic estimates (e.g. regression coefficient) AND variation (e.g. standard deviation) or associated estimates of uncertainty (e.g. confidence intervals)
☐	☒	For null hypothesis testing, the test statistic (e.g. *F*, *t*, *r*) with confidence intervals, effect sizes, degrees of freedom and *P* value noted<br>*Give P values as exact values whenever suitable.*
☒	☐	For Bayesian analysis, information on the choice of priors and Markov chain Monte Carlo settings
☒	☐	For hierarchical and complex designs, identification of the appropriate level for tests and full reporting of outcomes
☐	☒	Estimates of effect sizes (e.g. Cohen's *d*, Pearson's *r*), indicating how they were calculated

Our web collection on statistics for biologists contains articles on many of the points above.

Software and code

Policy information about availability of computer code

Data collection	Public clinical data analyzed are available at:<br>https://github.com/PangeaResearch/enlight-data<br>The PDXE models are available as Supplementary Material:<br>https://www.nature.com/articles/nm.3954<br>The TCGA data is available:<br>https://portal.gdc.cancer.gov/<br>The GTEX data:<br>https://www.gtexportal.org/home/
Data analysis	Custom code was published in the github page (https://github.com/xushiabbvie/TCGADEPMAP).<br>Data analysis packages used in the manuscript:<br>ggpubr 0.4.0 (R package)<br>ComplexHeatmap 2.6.2 (R package)<br>pROC 1.18.0 (R package)<br>FlowJo 10.8.1 (flow cytometry

For manuscripts utilizing custom algorithms or software that are central to the research but not yet described in published literature, software must be made available to editors and reviewers. We strongly encourage code deposition in a community repository (e.g. GitHub). See the Nature Portfolio guidelines for submitting code & software for further information.

Data

Policy information about availability of data

All manuscripts must include a data availability statement. This statement should provide the following information, where applicable:

- Accession codes, unique identifiers, or web links for publicly available datasets
- A description of any restrictions on data availability
- For clinical datasets or third party data, please ensure that the statement adheres to our policy

All data generated was shared as Supplementary Material or submitted to Figshare:
https://figshare.com/projects/TCGADEPMAP_Mapping_Translational_Dependencies_and_Synthetic_Lethalities_within_The_Cancer_Genome_Atlas/130193

Public clinical data analyzed are available at:
https://github.com/PangeaResearch/enlight-data
The PDXE models are available as Supplementary Material:
https://www.nature.com/articles/nm.3954
The TCGA data is available:
https://portal.gdc.cancer.gov/
The GTEX data:
https://www.gtexportal.org/home/

Research involving human participants, their data, or biological material

Policy information about studies with human participants or human data. See also policy information about sex, gender (identity/presentation), and sexual orientation and race, ethnicity and racism.

Reporting on sex and gender	NA
Reporting on race, ethnicity, or other socially relevant groupings	NA
Population characteristics	NA
Recruitment	NA
Ethics oversight	NA

Note that full information on the approval of the study protocol must also be provided in the manuscript.

Field-specific reporting

Please select the one below that is the best fit for your research. If you are not sure, read the appropriate sections before making your selection.

☒ Life sciences ☐ Behavioural & social sciences ☐ Ecological, evolutionary & environmental sciences

For a reference copy of the document with all sections, see nature.com/documents/nr-reporting-summary-flat.pdf

Life sciences study design

All studies must disclose on these points even when the disclosure is negative.

Sample size

No statistical methods were used to pre-determine sample sizes but our sample sizes are similar to those reported in previous publications that have tested tumor vulnerabilities and synthetic lethalities.
As a reference for CRISPR screens and animal studies for example:
1. Parrish, P. C. R. et al. Discovery of synthetic lethal and tumor suppressor paralog pairs in the human genome. Cell Rep 36, 109597 (2021).
2. Dede, M., McLaughlin, M., Kim, E. & Hart, T. Multiplex enCas12a screens detect functional buffering among paralogs otherwise masked in monogenic Cas9 knockout screens. Genome Biol 21, 262 (2020).
3.Neggers, J. E. et al. Synthetic Lethal Interaction between the ESCRT Paralog Enzymes VPS4A and VPS4B in Cancers Harboring Loss of Chromosome 18q or 16q. Cell Rep. 33, 108493 (2020).
4..Ogiwara, H. et al. Targeting p300 Addiction in CBP-Deficient Cancers Causes Synthetic Lethality by Apoptotic Cell Death due to Abrogation of MYC Expression. Cancer Discov 6, 430–445 (2016).
5. Charan, J. & Kantharia, N. D. How to calculate sample size in animal studies? J. Pharmacol. Pharmacother. 4, 303–306 (2013).

Data exclusions	No data was excluded from the synthetic lethality screens.
Replication	The replication was done using both AsCas12a and enAsCas12a across 14 different cell lines and showed very high correlation between the replicates.
Randomization	In this experiment there was no randomization. We followed similar experimental designs as the experiments cited above. Because of the smaller sample size of the animal experiments it would have been challenging to control for possible covariates.
Blinding	The investigators were not blinded to allocation during experiments and outcome assessment.

Reporting for specific materials, systems and methods

We require information from authors about some types of materials, experimental systems and methods used in many studies. Here, indicate whether each material, system or method listed is relevant to your study. If you are not sure if a list item applies to your research, read the appropriate section before selecting a response.

Materials & experimental systems

n/a	Involved in the study	
☐	☒	Antibodies
☐	☒	Eukaryotic cell lines
☒	☐	Palaeontology and archaeology
☐	☒	Animals and other organisms
☐	☒	Clinical data
☒	☐	Dual use research of concern
☒	☐	Plants

Methods

n/a	Involved in the study	
☒	☐	ChIP-seq
☐	☒	Flow cytometry
☒	☐	MRI-based neuroimaging

Antibodies

Antibodies used	Protein expression was quantified by Simple Western (ProteinSimple, BioTechne) using the following antibodies; PAPSS1 clone 1F4 (Abnova, H00009061-M05) at 1:100, PAPSS2 (Cell Signaling Technology (CST), #70638) at 1:50, PTEN (CST, #9552) at 1:100, CNOT7 (Santa Cruz, #sc-101009) at 1:10, CNOT8 (LSBio, #LS-C99242-400) at 1:1000 with beta-Actin clone 8H10D10 (CST, #3700), 1:1000 GAPDH clone 14C10 (CST, #2118) or 1:1000 alpha-Tubulin (CST, #2144) as loading controls. Flow cytometry analysis of sulfonated HSPGs was performed with 10E4 antibody conjugated to FITC and used at 1:200 (USBiological Life Sciences, #H1890-10)
Validation	Antibodies were validated by CRISPR knockout and data presented in Extended Figure 3J (CNOT7/CNOT8) and Extended Figure 4I-J (PAPSS1, PAPSS2). PTEN antibody specificity was confirmed in UMUC3 cells which carry endogenous deep deletion of PTEN and PAPSS2 (Extended Figure 4H). Specificity of antibody clone 10E4 (https://www.usbio.net/antibodies/H1890-10/Heparan%20Sulfate/data-sheet) for detection of sulfated Heparan Sulfate proteoglycans (HSPGs) was confirmed by enzymatic digestion with bacterial Heparinase III (shown in Figure 5D).

Eukaryotic cell lines

Policy information about cell lines and Sex and Gender in Research

Cell line source(s)	All cell lines were acquired from ATCC except PC3M (MD Anderson), HSC2 and HSC3 (JCRB).
Authentication	Cell line identity was confirmed by IDEXX STR testing.
Mycoplasma contamination	Cell lines were confirmed Mycoplasma negative by IDEXX mycoplasma testing.
Commonly misidentified lines (See ICLAC register)	None of the cell lines used are part of the ICLAC v12 registry.

Animals and other research organisms

Policy information about studies involving animals; ARRIVE guidelines recommended for reporting animal research, and Sex and Gender in Research

Laboratory animals	Mouse, CB17/SCID and Mouse, SCID/Beige were used in this study (Charles River).
Wild animals	N/A
Reporting on sex	N/A
Field-collected samples	N/A

Ethics oversight	In vivo experiments were conducted in compliance with AbbVie's Institutional Animal Care and Use Committee and the NIH guidelines in the Health Guide for Care and Use of Laboratory Animals. Tumor measurements of length (L) and width (W) were obtained using calipers and volume (V) calculated using the formula V=(LxW2)/2. A maximum of 2000 mm3 tumor volume was allowed as per institutional guidelines.

Note that full information on the approval of the study protocol must also be provided in the manuscript.

Clinical data

Policy information about clinical studies

All manuscripts should comply with the ICMJE guidelines for publication of clinical research and a completed CONSORT checklist must be included with all submissions.

Clinical trial registration	We have only analyzed public clinical data generated by others. The list of datasets are available and protocols were published in earlier publications (see: https://github.com/PangeaResearch/enlight-data)
Study protocol	*Note where the full trial protocol can be accessed OR if not available, explain why.*
Data collection	*Describe the settings and locales of data collection, noting the time periods of recruitment and data collection.*
Outcomes	*Describe how you pre-defined primary and secondary outcome measures and how you assessed these measures.*

Flow Cytometry

Plots

Confirm that:

☒ The axis labels state the marker and fluorochrome used (e.g. CD4-FITC).

☒ The axis scales are clearly visible. Include numbers along axes only for bottom left plot of group (a 'group' is an analysis of identical markers).

☒ All plots are contour plots with outliers or pseudocolor plots.

☒ A numerical value for number of cells or percentage (with statistics) is provided.

Methodology

Sample preparation	Adherent cell lines were harvested and single-cell suspended by standard techniques (trypsinization).
Instrument	BD LSRFortessa X-20, Model no. 658226R1
Software	Flowjo v10.8.1
Cell population abundance	No FACS sorting was performed.
Gating strategy	Cells were first gated by FSC-A/SSC-A (~95%) and single cells by FSC-A/FSC-H (~98%). DAPI staining was used to gate viable cells (~98%). Unstained cells and/or Heparinase III treated cells were used for establishing the 10E4-FITC positive gates. Gating strategy is shown in Extended Figure 8.

☒ Tick this box to confirm that a figure exemplifying the gating strategy is provided in the Supplementary Information.
