# Supplementary figures and images for: Building a translational cancer dependency map for The Cancer Genome Atlas
Source: Nat Cancer. 2024 Jul 15;5(8):1176–94. doi: 10.1038/s43018-024-00789-y (PMC11358024; doi:10.1038/s43018-024-00789-y)

Figure 4 H. Uncropped photographs

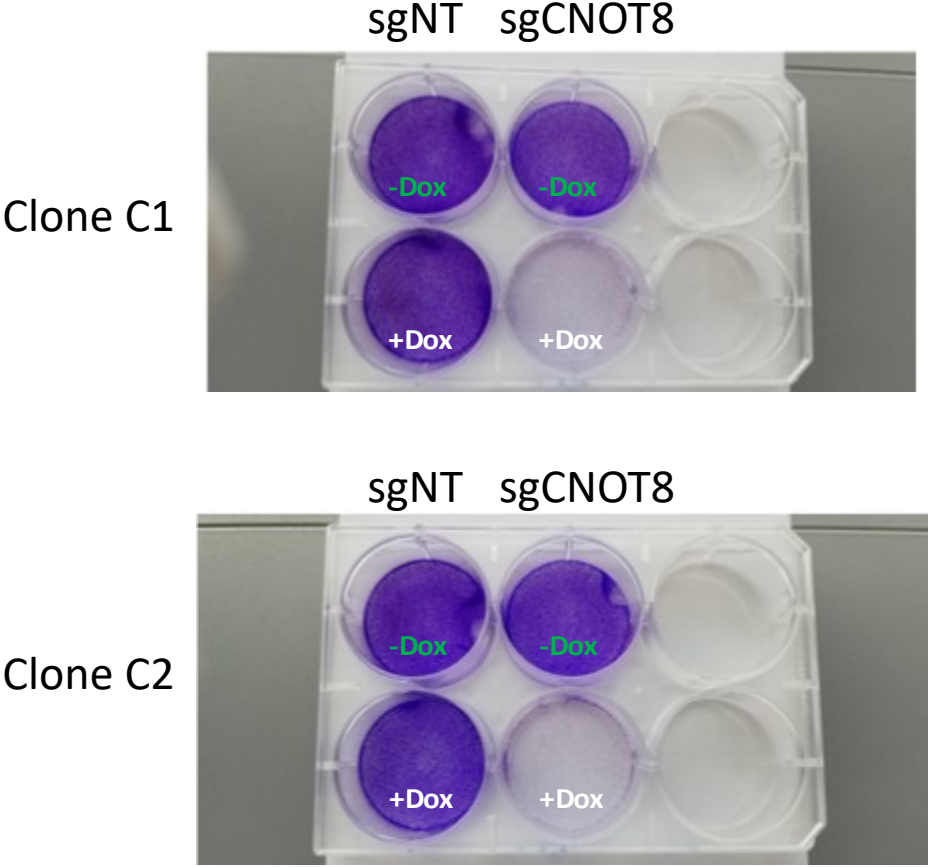

Supplement: Supplementary file 13 — Uncropped image for Fig. 4h. [file 43018_2024_789_MOESM13_ESM.pdf]
