# Supplementary material for: Building a translational cancer dependency map for The Cancer Genome Atlas
Source: Nat Cancer. 2024 Jul 15;5(8):1176–94. doi: 10.1038/s43018-024-00789-y (PMC11358024; doi:10.1038/s43018-024-00789-y)

Extended Figure 3J.

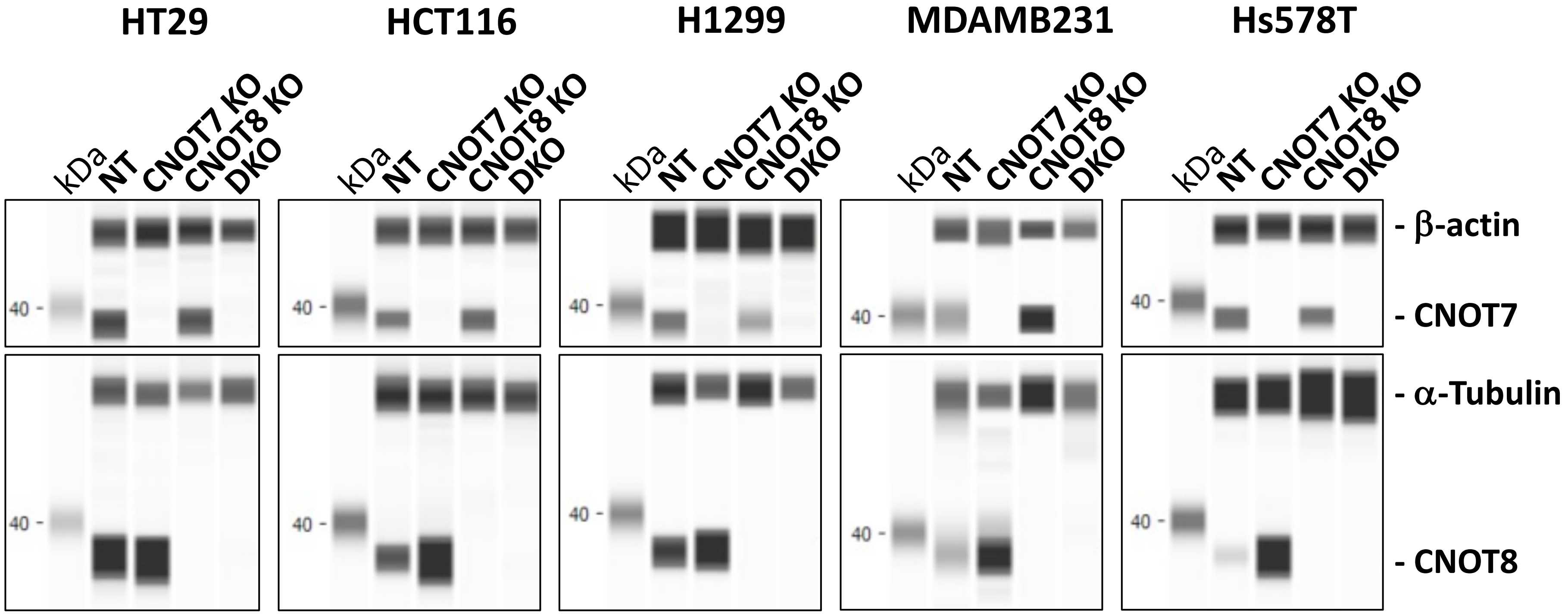

Extended Figure 3J – uncropped for source data: HT29

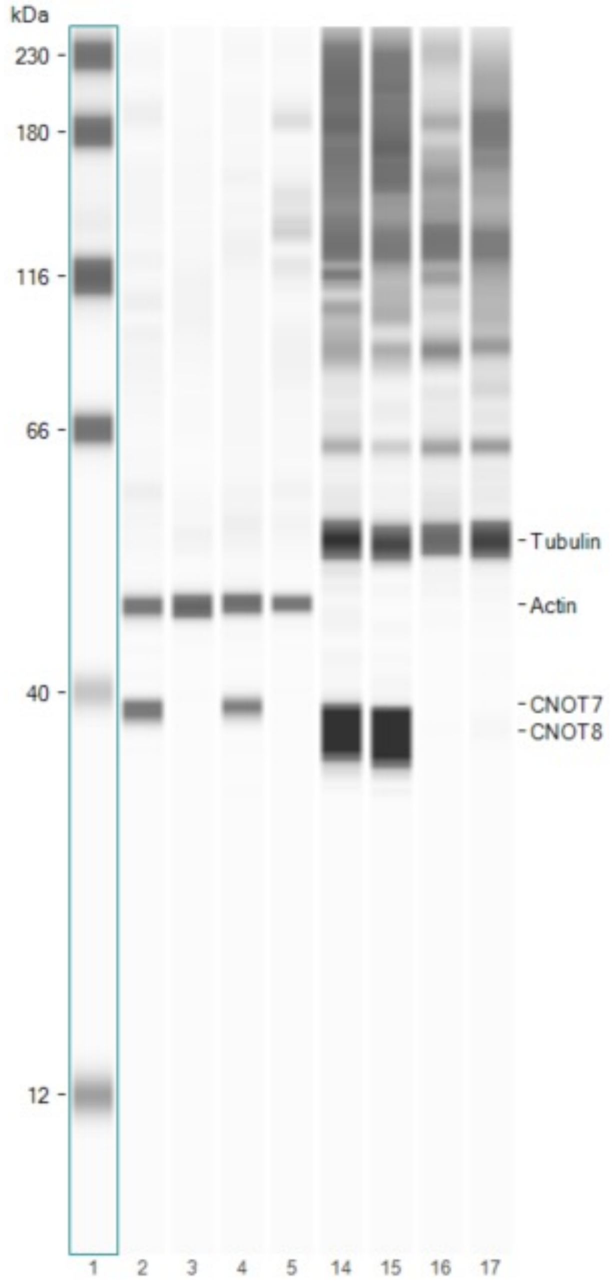

Extended Figure 3J – uncropped for source data: HCT116

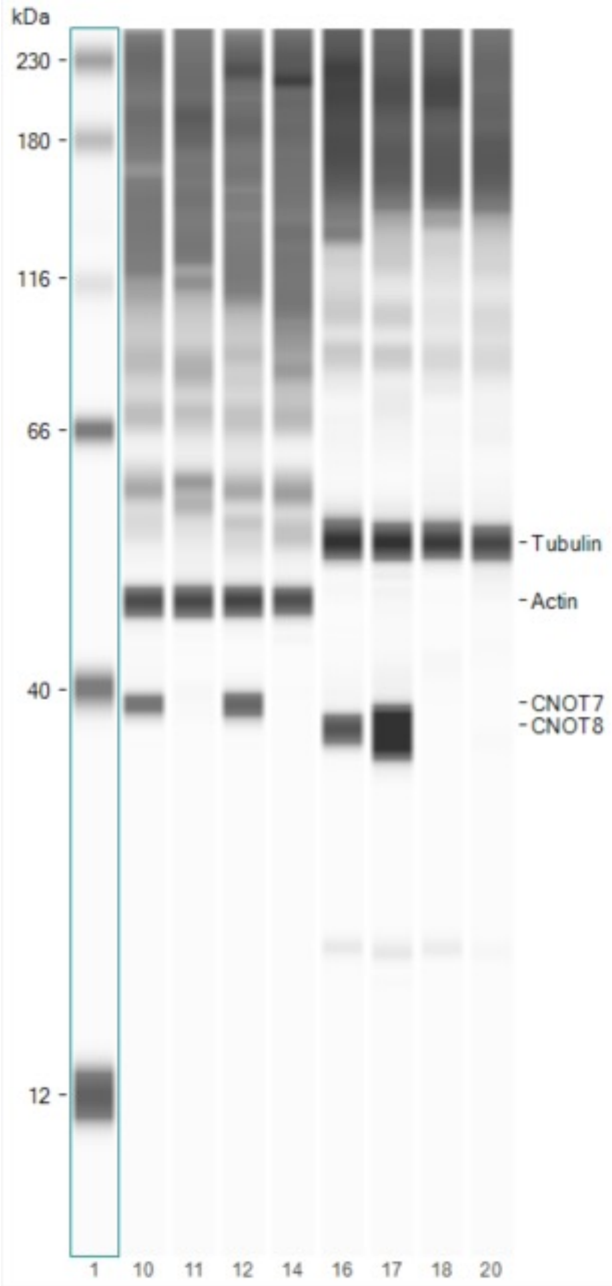

Extended Figure 3J – uncropped for source data: Hs578T

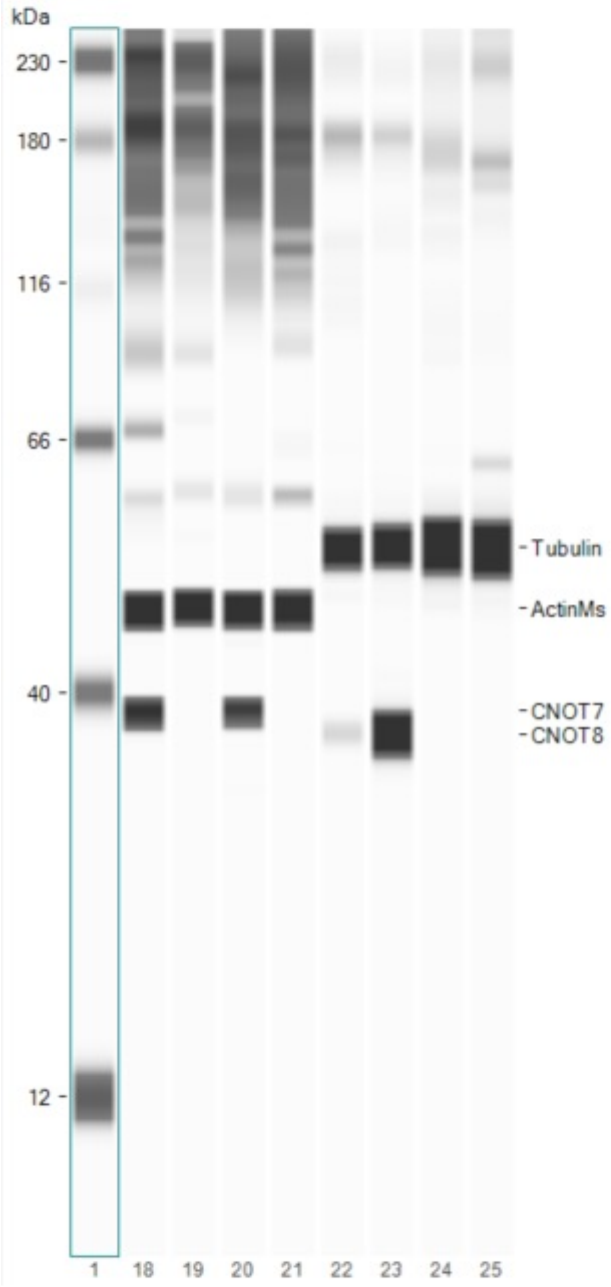

Extended Figure 3J – uncropped for source data: H1299

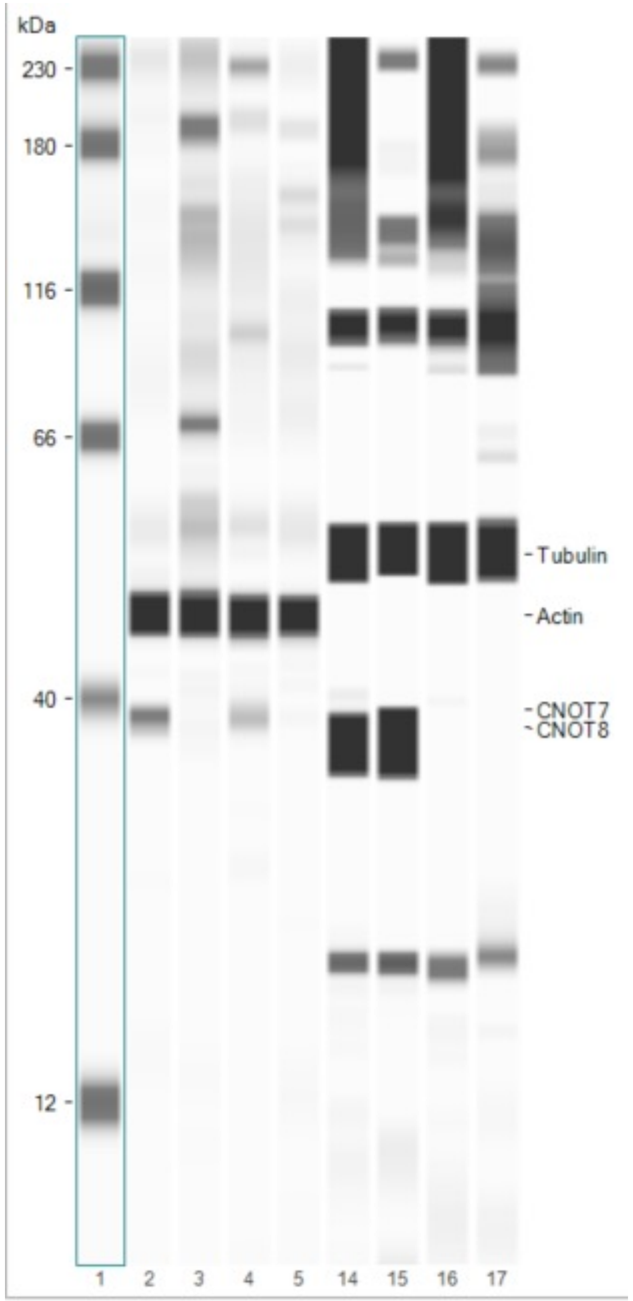

Extended Figure 3J – uncropped for source data: MDAMB231

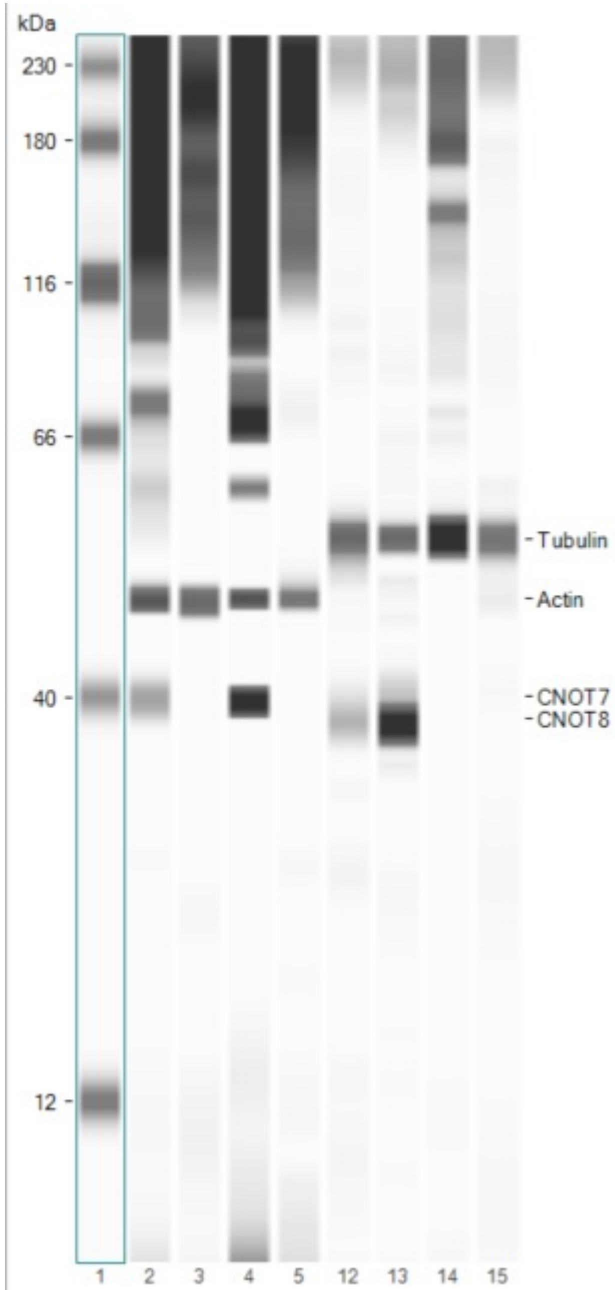

Extended Figure 3J – uncropped for source data: Hs578T

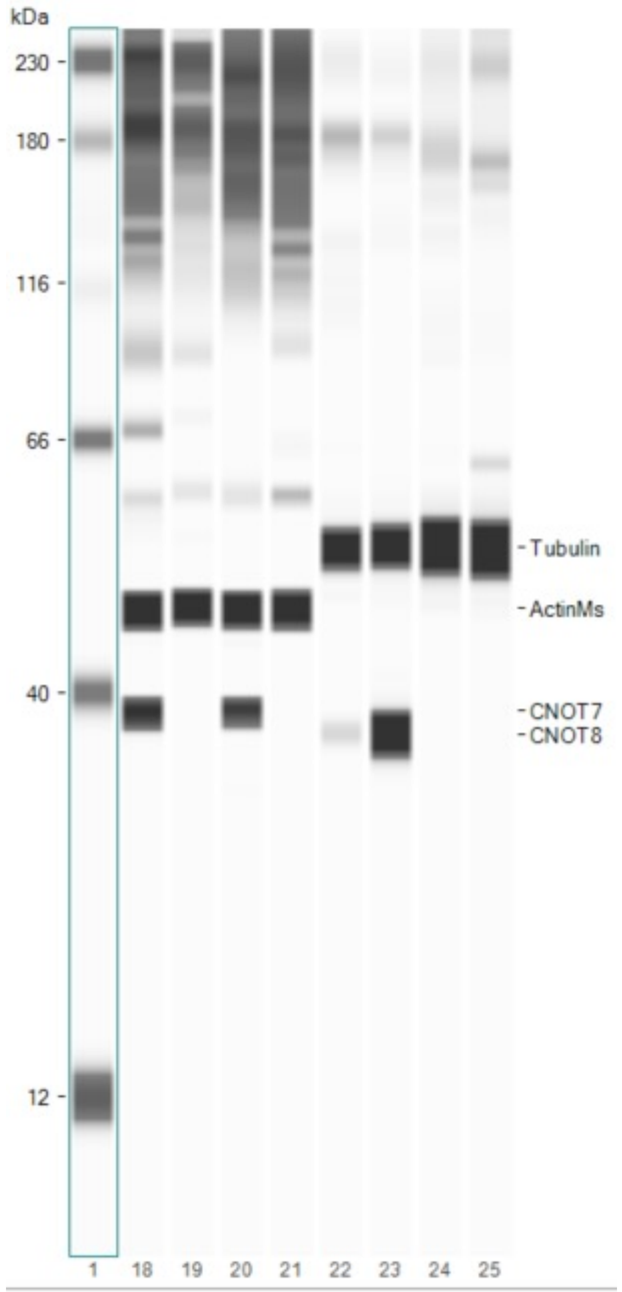

Updated Extended Figure 4H

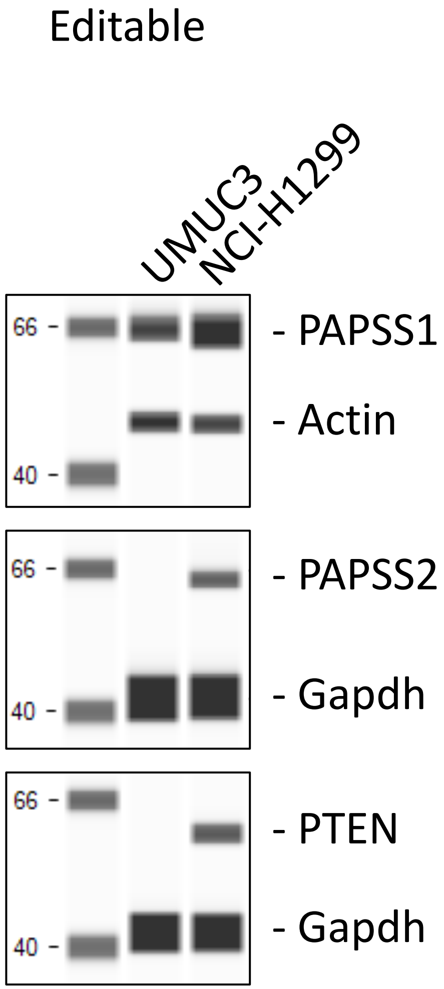

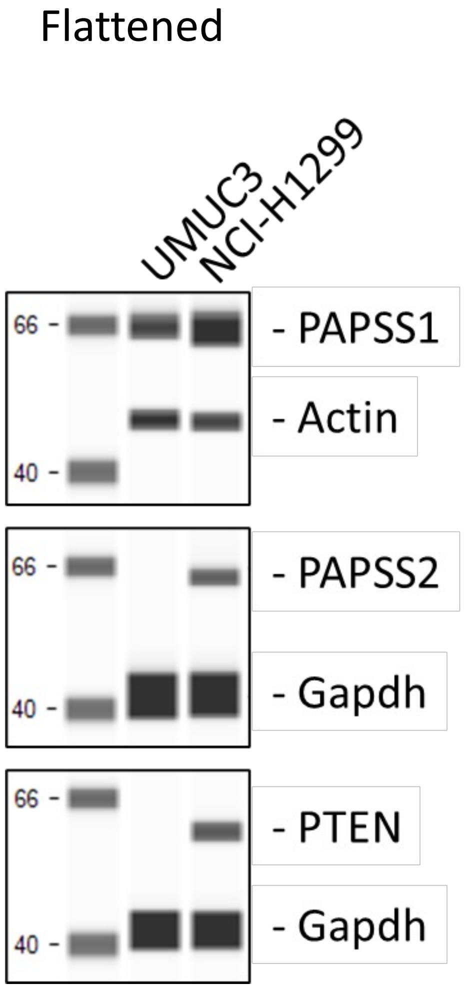

Extended Data Figure 4H – uncropped for source data

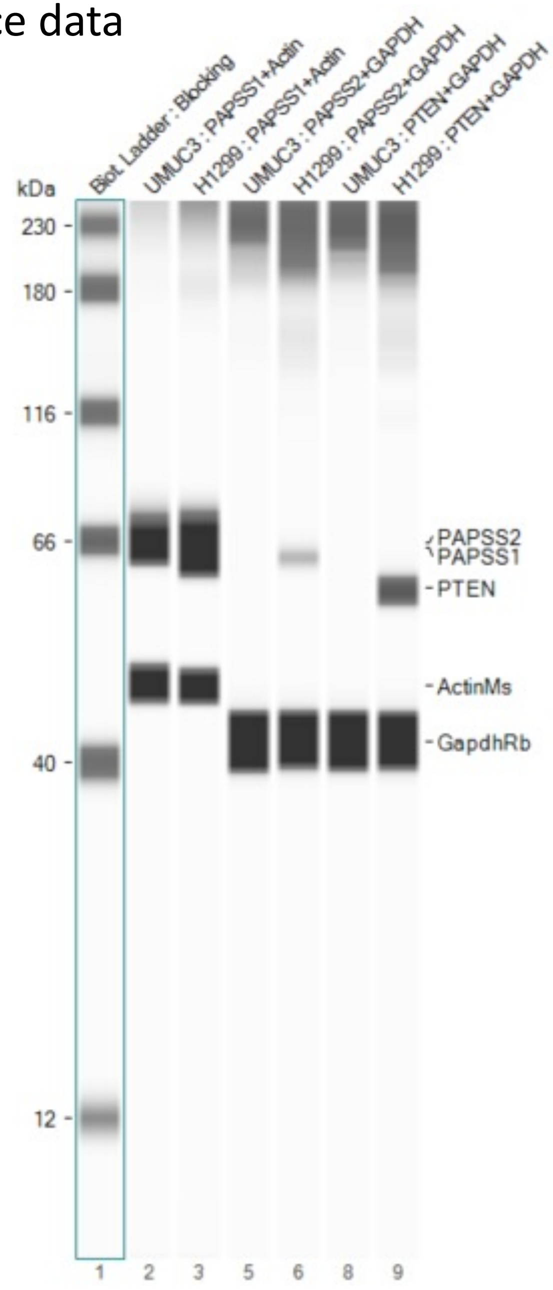

Extended Data Figure 4I – uncropped for source data

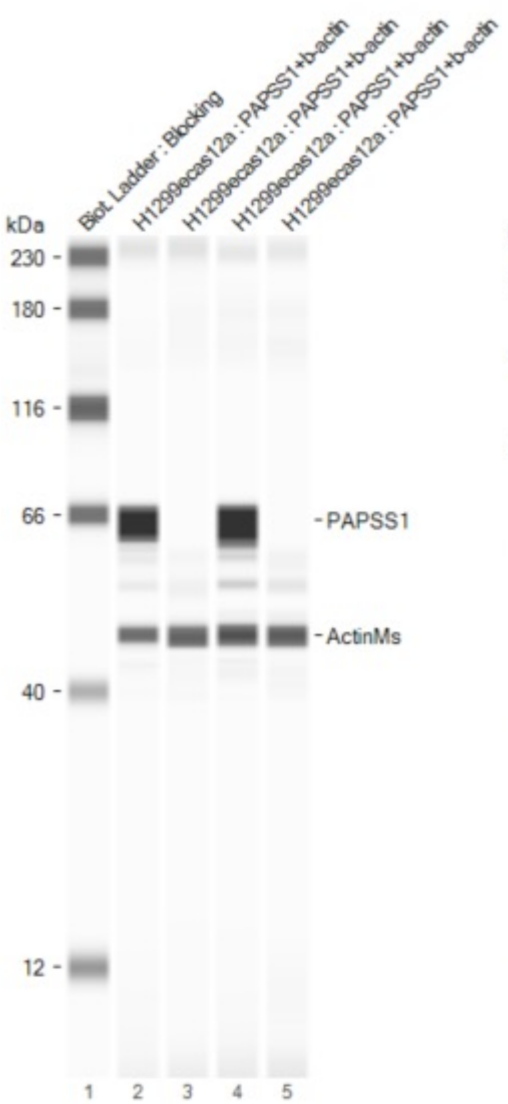

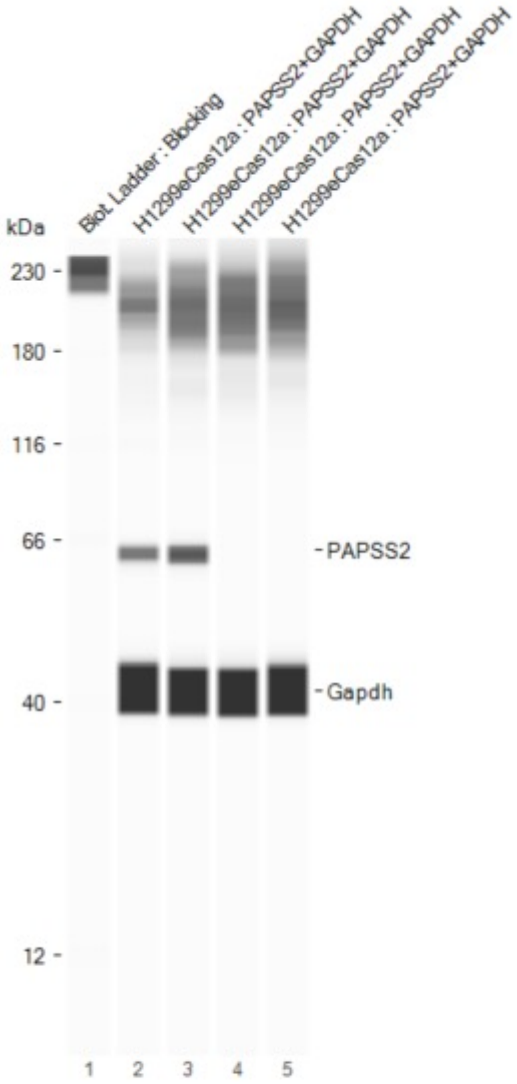

Extended Data Figure 4J – uncropped for source data

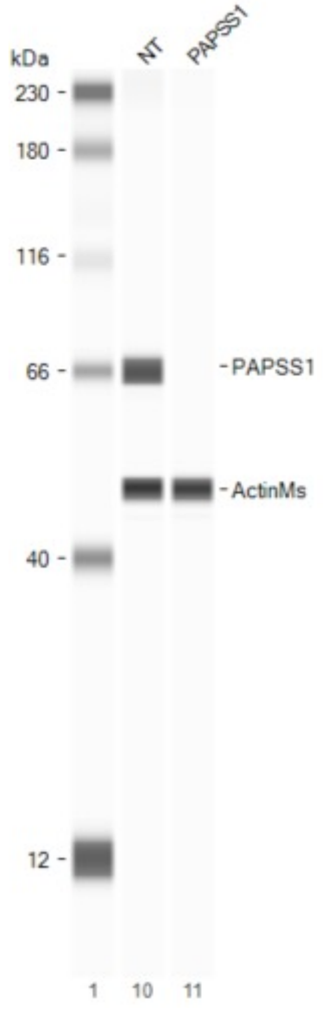

Extended Data Figure 4K – uncropped for source data

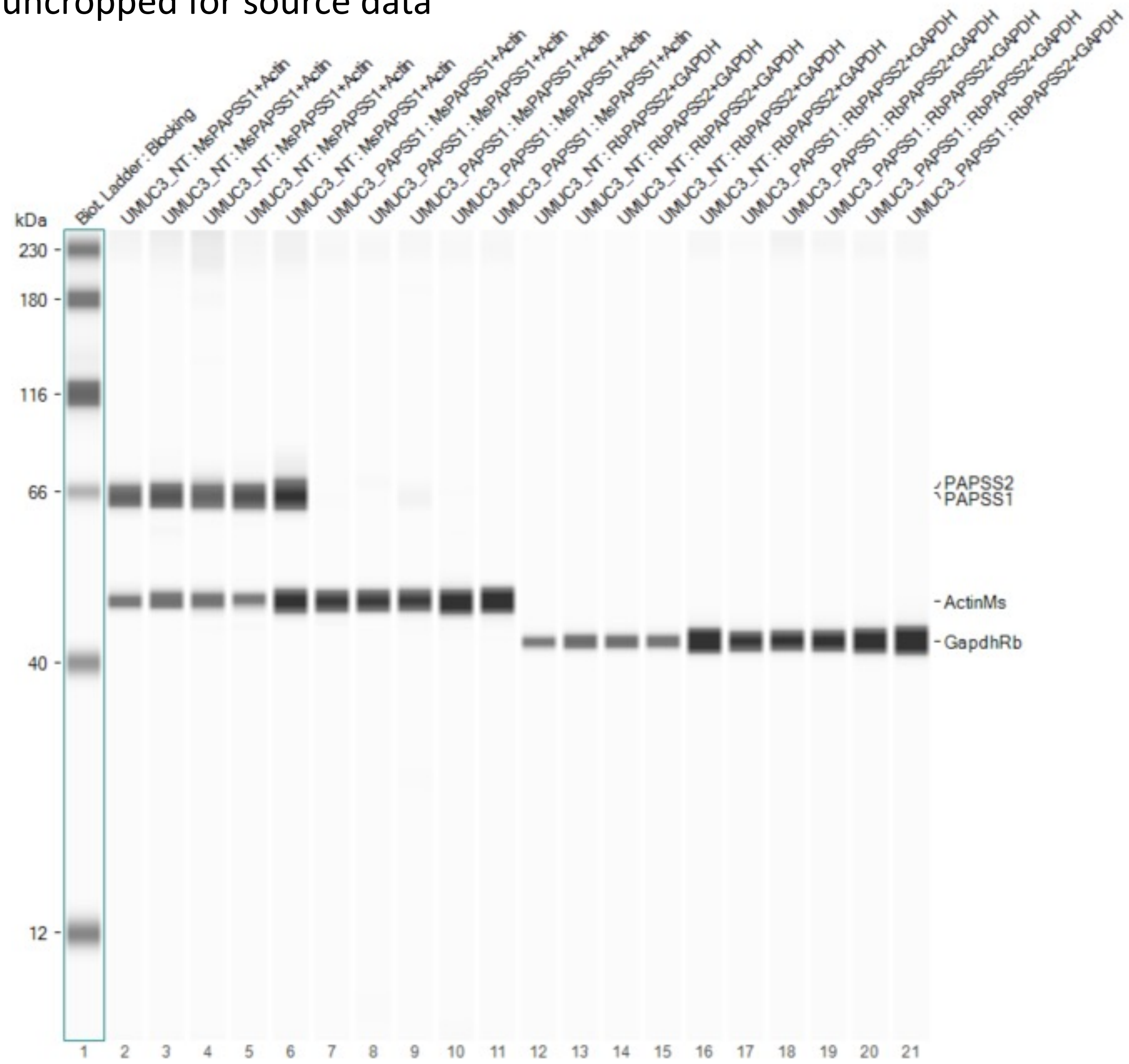

Supplement: Supplementary file 23 — Uncropped images for Extended Data Fig. 10. [file 43018_2024_789_MOESM23_ESM.pdf]
